# An influenza HA stalk reactive polymeric IgA antibody exhibits anti-viral function regulated by binary interaction between HA and the antibody

**DOI:** 10.1371/journal.pone.0245244

**Published:** 2021-01-07

**Authors:** Kaori Sano, Shinji Saito, Tadaki Suzuki, Osamu Kotani, Akira Ainai, Elly van Riet, Koshiro Tabata, Kumpei Saito, Yoshimasa Takahashi, Masaru Yokoyama, Hironori Sato, Takahiro Maruno, Kaede Usami, Susumu Uchiyama, Kiyoko Ogawa-Goto, Hideki Hasegawa

**Affiliations:** 1 Department of Pathology, National Institute of Infectious Diseases, Shinjuku, Tokyo, Japan; 2 Division of Infectious Diseases Pathology, Department of Global Infectious Diseases, Tohoku Graduate School of Medicine, Sendai, Miyagi, Japan; 3 Influenza Virus Research Center, National Institute of Infectious Diseases, Musashimurayama, Tokyo, Japan; 4 Pathogen Genomics Center, National Institute of Infectious Diseases, Musashimurayama, Tokyo, Japan; 5 Department of Immunology, National Institute of Infectious Diseases, Shinjuku, Tokyo, Japan; 6 Department of Biotechnology, Graduate School of Engineering, Osaka University, Suita, Osaka, Japan; 7 Exploratory Research Center on Life and Living Systems (ExCELLS), National Institutes of Natural Sciences, Okazaki, Aichi, Japan; 8 Nippi Research Institute of Biomatrix, Nippi Incorporated, Toride, Ibaraki, Japan; 9 Global Virus Network, Baltimore, MD, United States of America; University of South Dakota, UNITED STATES

## Abstract

IgA antibodies, which are secreted onto the mucosal surface as secretory IgA antibodies (SIgAs), play an important role in preventing influenza virus infection. A recent study reported that anti-hemagglutinin (HA) head-targeting antibodies increase anti-viral functions such as hemagglutination inhibition (HI) and virus neutralization (NT), in addition to HA binding activity (reactivity) via IgA polymerization. However, the functional properties of anti-viral IgA antibodies with mechanisms of action distinct from those of anti-HA head-targeting antibodies remain elusive. Here, we characterized the functional properties of IgG, monomeric IgA, and polymeric IgA anti-HA stalk-binding clones F11 and FI6, and B12 (a low affinity anti-HA stalk clone), as well as Fab-deficient (ΔFab) IgA antibodies. We found that IgA polymerization impacts the functional properties of anti-HA stalk antibodies. Unlike anti-HA head antibodies, the anti-viral functions of anti-HA stalk antibodies were not simply enhanced by IgA polymerization. The data suggest that two modes of binding (Fab paratope-mediated binding to the HA stalk, and IgA Fc glycan-mediated binding to the HA receptor binding site (RBS)) occur during interaction between anti-stalk HA IgA antibodies and HA. In situations where Fab paratope-mediated binding to the HA stalk exceeded IgA Fc glycan-mediated binding to HA RBS, IgA polymerization increased anti-viral functions. By contrast, when IgA Fc glycan-mediated binding to the HA RBS was dominant, anti-viral activity will fall upon IgA polymerization. In summary, the results suggest that coordination between these two independent binding modules determines whether IgA polymerization has a negative or positive effect on the anti-viral functions of anti-HA stalk IgA antibodies.

## Introduction

Influenza is a highly contagious infectious disease caused by the influenza virus. The main targets of this virus in humans are epithelial cells lining the upper respiratory tract. Therefore, IgA antibodies, which are secreted onto the mucosal surface as secretory IgA antibodies (SIgAs), play an important role in preventing influenza virus infection [1–3]. Currently available influenza vaccines are of the injectable type, which induce systemic IgG responses but not mucosal IgA responses; such vaccines mitigate the severity of disease following virus infection but cannot prevent infection in the first place [4]. By contrast, intranasally-administered inactivated vaccines, which are currently under development, induce secretion of SIgAs by respiratory mucosal tissues [5,6]. Thus, intranasal inactivated influenza vaccines are promising vaccine candidates that could protect humans from infection by influenza virus [7,8].

According to a study based on human polyclonal SIgAs isolated from nasal wash samples obtained from intranasal inactivated influenza vaccine recipients, SIgAs are secreted by the upper respiratory tract mucosa in the form of monomers, dimers, and polymers (which are larger than dimers). Studies show that SIgA polymers exhibit higher virus-neutralizing activity than dimers or monomers [9,10]. However, the detailed molecular mechanism(s) that underpins this phenomenon remains unclear. Previously, Saito *et al*. developed a method of generating polymeric SIgA *in vitro*, which involves co-transfecting mammalian cells with expression plasmids encoding the four components of SIgA: immunoglobulin heavy chain (HC), light chain (LC), joining chain (JC), and the secretory component (SC) [11]. SIgAs generated using this method are useful in that they can be used to evaluate the effect of SIgA polymerization by comparing the anti-viral activities of monomeric and polymeric SIgAs harboring identical variable regions. This method was used to show that anti-HA head-targeting antibodies have increased anti-viral activity, including HA binding activity (reactivity), hemagglutination inhibition (HI) activity, and virus neutralization (NT) activity upon IgA polymerization [11]. However, the effects of IgA polymerization on the anti-viral functions of antibodies that target the influenza HA stalk region remain unexplained; these antibodies play a beneficial role in protecting against influenza virus infection through mechanisms different from those of anti-HA head-targeting antibodies.

The HA stalk is a relatively well-conserved region among multiple strains and subtypes of influenza virus [12]. Thus, a large proportion of influenza virus broadly neutralizing antibodies (bnAbs) identified to date are anti-HA stalk antibodies [13–16]. Some studies have attempted to induce anti-HA stalk antibodies via vaccination, and to show that these antibodies confer protection against a broad range of influenza virus infections [17–20]. Anti-HA stalk bnAbs inhibit conformational changes in HA molecules, a process essential for fusion of the virus with the cell membrane following virus attachment [21–23]; by contrast, anti-HA head bnAbs target the receptor binding domain, thereby inhibiting virus attachment to target cells [24,25]. In addition, anti-HA stalk antibodies possess neuraminidase (NA) inhibition (NI) activity [26–28]. NA digests cell surface sialic acids that bind to the HA molecule of newly budding viruses to release viral progeny from the cell surface. By binding to the HA stalk, anti-HA stalk antibodies physically impede access to neighboring NA molecules, a process that is required for sialic acid digestion; this results in NI activity due to steric hindrance [26]. Thus, a detailed understanding of the anti-viral humoral immune mechanisms that fight influenza viruses is critical for development of more effective treatments and prophylactic strategies. However, no reports have examined anti-HA stalk antibodies in recipients of intranasal inactivated influenza vaccines, or anti-viral mechanisms that operate in the upper respiratory mucosa, which is the first line of defense against respiratory pathogens. Therefore, to better understand the mechanisms underlying mucosal humoral immunity induced by intranasal inactivated influenza vaccination, as well as the functional traits of mucosal IgA antibodies, it is essential to verify the presence of anti-HA stalk antibodies in recipients of intranasal inactivated influenza vaccines and to understand the effects of SIgA polymerization on these antibodies.

In this study, we isolated an anti-HA stalk antibody from a recipient of an intranasal inactivated influenza vaccine and examined the effects of IgA polymerization on its anti-viral activity. The results suggest that IgA polymerization impacts the functions of anti-HA stalk antibodies in various ways depending on the mode of interaction between the antibody and viral glycoproteins.

## Results

### Anti-viral activity of the HA stalk IgG antibody and of IgA monomers and polymers

To examine the effect of IgA polymerization on anti-HA stalk antibody functions, we compared the anti-viral activity of anti-HA stalk IgG with that of IgA monomers and polymers. First, we isolated the human antibody clone F11 (an anti-HA stalk antibody clone) from a recipient of an intranasal inactivated influenza vaccine. Detailed information regarding the procedure used to screen clone F11 is provided in S1 and S2 Tables. Characterization of antibody clone F11 is described in S1 and S2 Figs and S3 Table. In addition to clone F11, we used a previously reported anti-HA stalk bnAb, FI6 [29], for these comparisons. For each antibody clone, we generated IgA monomers and IgA polymers comprising the two subclasses of IgA: IgA1 and IgA2 (allotype IgA2m2). IgA polymers were produced using methods established previously [11], and successful production of IgA polymers of antibody clones F11 and FI6 was confirmed by SDS-PAGE (S3D Fig), HPLC (S3B Fig), and sedimentation velocity analytical ultracentrifugation (SV-AUC; S3E–S3I Fig) analyses.

Anti-viral activity of F11 and FI6 IgG1, and monomeric or polymeric IgA1 and IgA2m2 (including NT activity, HI activity, NI activity, and binding activity), was tested against virus strains A/California/7/2009 (H1N1, Cal7), A/New Caledonia/20/1999 (H1N1, NC20), A/Puerto Rico/8/1934 (H1N1, PR8), A/Victoria/210/2009 (H3N2, Vic210), and A/New York/55/2004 (H3N2, NY55). The results of these assays are summarized in Table 1.

**Table 1:** Anti-viral functions of IgG and IgA antibodies derived from clones F11 and FI6.

		F11					FI6				
		H1N1			H3N2		H1N1			H3N2	
		Cal7	NC20	PR8	Vic210	NY55	Cal7	NC20	PR8	Vic210	NY55
**NT activity** [μg/mL] Geometric mean (geometric SD)	G	11.2 (1.69)	89.1 (1.33)	83.3 (1.79)	>500	>500	5.00 (1)	31.5 (1.43)	28.1 (1.69)	2.19 (1.69)	3.48 (1.33)
	A1M	2.81 (2.25)	28.1 (1.69)	50.0 (1.55)	1.74 (1.98)	11.1 (1.79)	3.15 (1.76)	6.25 (1)	39.7 (2.05)	0.137 (1.33)	0.69 (2.07)
	A1P	4.45 (1.69)	15.8 (1.43)	25.0 (1) *	1.55 (1.43)	7.81 (1.55)	1.98 (1.76)	4.96 (1.43)	11.1 (1.33) *	0.154 (2.32)	0.775 (1.76)
	A2M	4.45 (2.25)	11.1 (1.33)	89.1 (1.33)	0.548 (1.69)	3.1 (1.76)	2.5 (2.14)	8.84 (1.46)	31.5 (1.43)	0.345 (1.79)	1.10 (1.98)
	A2P	7.07 (1.46)	5.57 (1.33) *	50.0 (1) *	0.194 (1.43) *	0.977 (1.55) **	0.88 (1.46) *	1.75 (1.98) **	6.25 (1) **	0.0308 (1.56) **	0.217 (1.69) **
**HI activity** [μg/mL] Geometric mean (geometric SD)	G	>500	>500	>500	>500	>500	>500	>500	>500	>500	>500
	A1M	62.5 (1)	62.5 (1)	354 (1.46)	9.84 (1.43)	15.6 (1)	177 (1.46)	7.81 (1)	500 (1)	1.95 (1)	2.76 (1.46)
	A1P	62.5 (1)	62.5 (1)	500 (1)	19.7 (1.43) *	27.8 (1.33) *	88.4 (1.46) *	15.6 (1) **	>500	1.95 (1)	3.48 (1.33)
	A2M	88.4 (1.46)	31.3 (1)	500 (1)	7.81 (1)	7.81 (1)	125 (1)	11.1 (1.46)	500 (1)	3.91 (1)	3.91 (1)
	A2P	49.6 (1.76)	7.81 (1) **	500 (1)	2.46 (1.43) **	3.48 (1.33) **	44.2 (1.46) **	11.1 (1.46)	250 (1) **	0.691 (1.46) **	0.775 (1.43) **
**NI activity** [ng/mL] (EC_50_)	G	1616	2136	3315	ND	ND	551	610	1195	96.1	64.2
	A1M	267	148	301	192	125	117	106	165	126	48.9
	A1P	40.6 ****	106 **	222	264 *	187 *	69.5 ***	48.3 ****	149	107 *	64.1
	A2M	215	61.7	683	927	546	57.4	11.4	173	23.2	12.7
	A2P	30.4 ****	16.2 ****	418 **	258 ***	121 **	114.9 ***	14.02 *	302 ***	6.65 ****	4.27 ***
**Binding activity** [ng/mL] (EC_50_)	G	800	138	422	2.51+06	8.49E+07	291	59.5	42.5	2.45E+05	1.11E+04
	A1M	492	1.16E+03	3.09E+03	1.95E+04	2.01E+03	305	602	571	2.43E+03	2.48E+03
	A1P	502	730 ****	1.21E+03 ****	6.51E+03 **	3.43E+03 ***	258 ****	549 *	758 ***	6.84E+03 ****	2.58E+03
	A2M	4.64E+04	1.35E+04	3.17E+04	1.90E+04	4.92E+03	1.16E+07	ND	8.45E+05	1.56E+03	647
	A2P	1.56E+04 ****	3.39E+04 ***	7.75E+04 ****	6.85E+03 ****	303 ****	5.70E+04 ****	9.39E+03 ****	1.45E+04 ****	3.69E+03 ****	3.06E+03 ****

NT activity, virus-neutralizing activity. Minimum virus-neutralizing concentrations are presented as the geometric mean (geometric SD) of six technical replicates. HI activity, hemagglutination inhibition activity. Minimum HI concentrations are presented as the geometric mean (geometric SD) of six technical replicates. NI activity, neuraminidase inhibition activity. EC_50_ values were calculated from three technical replicates. Binding activity: antibody binding to recombinant HA measured by ELISA. EC_50_ values were calculated from three technical replicates. G, IgG1; A1M, IgA1 monomer; A1P, IgA1 polymer; A2M, IgA2m2 monomer; A2P, IgA2m2 polymer; Cal7, A/California/7/2009 (H1N1); NC20, A/New Caledonia/20/1999 (H1N1); PR8, A/Puerto Rico/8/1934 (H1N1); Vic210, A/Victoria/210/2009 (H3N2); NY55, A/New York/55/2004 (H3N2, NY55); ND, not detected

*p < 0.05

**p < 0.01

***p < 0.001

****p < 0.0001 (Mann–Whitney test, comparing NT and HI activity of monomers with polymers; two-way ANOVA comparing NI and binding activity curves of monomers with those of polymers).

Increased NT activity due to IgA polymerization was more evident for IgA2m2 antibodies derived from clones FI6 and F11, both of which showed marked increases in NT titer against Cal7, NC20, and PR8 viruses; the exception was F11 IgA2m2 against Cal7. The NT activity of F11 and FI6 IgA1 against the PR8 virus was significantly higher after polymerization. In some cases, NT activity increased after converting the antibody backbone from IgG to an IgA1 or IgA2m2 monomer, suggesting that not only the number of Fabs per molecule but also differences in the antibody constant region contribute to the increased NT titer. This hypothesis was further supported by the results of NT assays using virus strains Vic210 and NY55. F11, which did not exhibit NT activity in the IgG state, did show NT activity when converted to IgA, clearly suggesting that anti-viral potency is derived from the IgA backbone. As described above, FI6 neutralized the H3 virus in both its IgG and IgA forms, although we observed a marked increase in NT activity after converting the IgG backbone to an IgA1 backbone (Table 1). These observations were consistent with those of a previous report in which enhancement in virus neutralization activity was observed after converting the IgG backbone of an anti-HA stalk antibody to an IgA backbone [29].

To verify whether the increase in NT induced by IgA polymerization was due simply to increased avidity, we used ELISAs to examine binding of IgA monomers and polymers to HA proteins derived from Cal7, NC20, PR8, Vic210, and NY55 viruses. However, we found no direct association between increased IgA binding activity and NT activity after IgA polymerization (Table 1), suggesting that the NT activity of anti-HA stalk IgA antibodies is not defined simply by the antibodies’ binding activity, but also by their other functions. This observation is inconsistent with those of a previous report of anti-HA head antibodies [11], suggesting that the impact of IgA polymerization does not have an equal positive or negative impact on the anti-viral functions of anti-HA stalk antibodies.

### Polymerization of anti-HA stalk IgA antibodies increases HI activity due to efficient masking of the HA receptor binding site (RBS)

**Fig 1:**
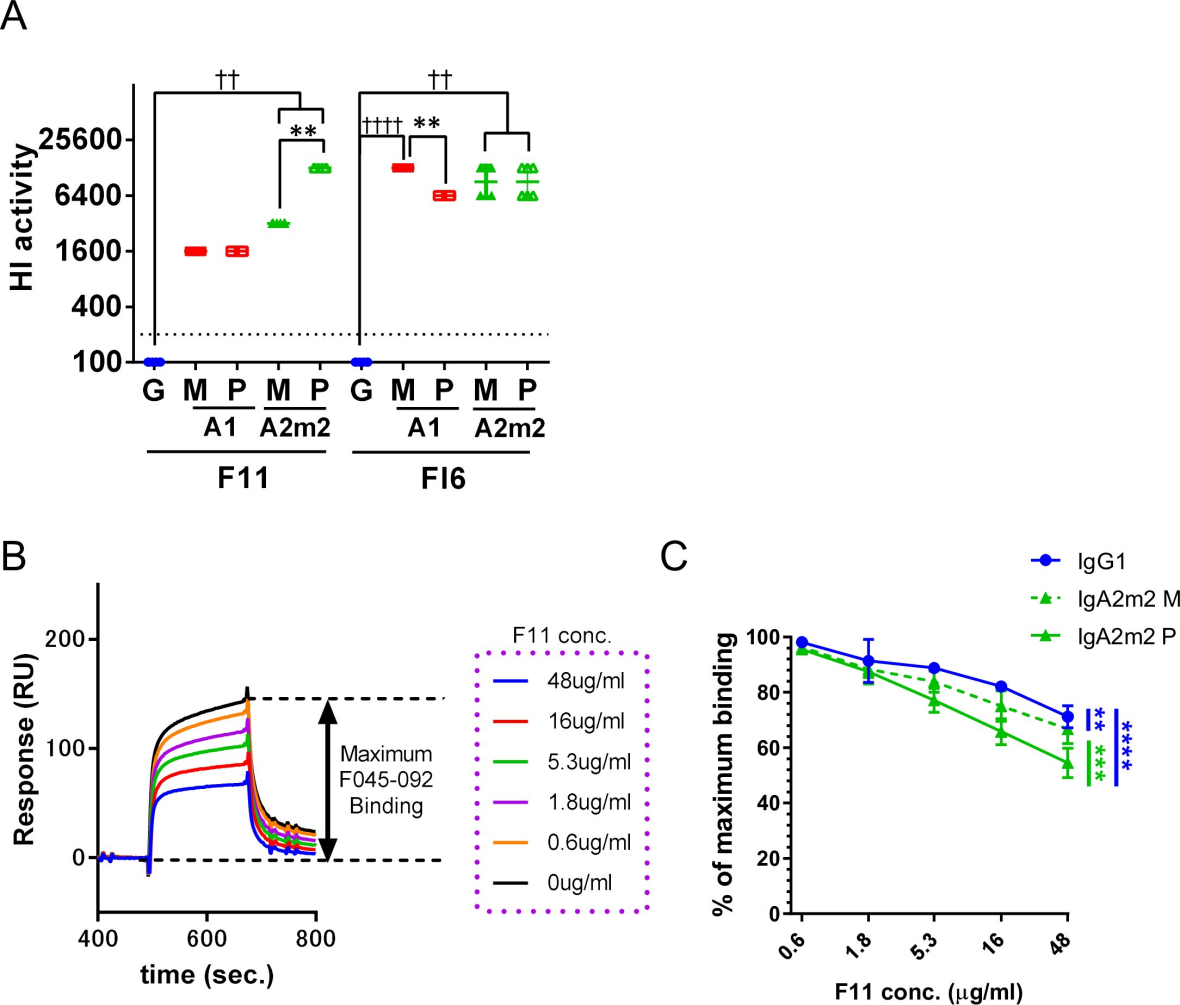
IgA polymers mask the hemagglutinin (HA) receptor binding site. (A) The HI titer for each IgG1, monomeric (M) or polymeric (P) IgA1 (A1), and IgA2m2 (A2m2) antibody derived from clones F11 and FI6, tested against virus strain A/New Caledonia/20/1999 (H1N1, NC20). HI activity is presented in the scatter plots as the geometric mean, with the geometric standard deviation (SD) of six technical replicates. Y-axis values represent the HI titer, calculated as the 100,000/minimum concentration (μg/ml), which represents HI activity. Dotted lines represent the detection limit (y = 200; 500 μg/mL). **p < 0.01, comparing monomers with polymers (Mann–Whitney U test). ☨☨*p* < 0.01 and ☨☨☨☨*p* < 0.0001, comparing IgA monomers/polymers with IgG (Kruskal–Wallis test, followed by Dunn’s multiple comparison test). For statistical analysis, a provisional minimum HI activity value (y = 100; 1000 μg/mL) was applied to samples in which the HI activity was below the detection limit. (B) Schematic diagram showing RBS blockade, as assessed by SPR. First, recombinant HA from virus strain A/New Caledonia/20/1999 (H1N1) was bound to IgG1 or to monomeric or polymeric F11 IgA2m2. The amount of F045-092 IgG1 binding to F11-pre-bound HA was then measured to evaluate RBS blocking by F11. The relative response (RU) was acquired immediately after addition of F045-092 IgG1 (binding stability). The maximum stability value was acquired by measuring the RU immediately following binding of F045-092 IgG1 to free HA. (C) Binding of F045-092 IgG1 to F11 IgG1-, IgA2m2-, or IgA2m2-pre-bound HA. Y-axis values represent the ratio of F045-092 IgG1 binding to the maximum stability value. **p < 0.01, ***p < 0.001, and ****p < 0.0001 (two-way ANOVA).

To identify factors other than avidity that determine the NT titer of anti-HA stalk antibodies, we examined other anti-viral functions of anti-HA stalk IgG, IgA monomers, and IgA polymers, starting with HI activity. HI activity is one of the main components of anti-influenza virus immunity *in vivo*, along with anti-viral activity conferred by cellular immunity [30] and antibodies targeting various sites of viral components [31]; traditionally, HI activity is the correlate of protection against influenza in humans. Antibodies with HI activity block interaction with the RBS located on the HA head [32]. Consistent with previous data, we found that F11, an HA stalk-binding antibody with an IgG1 backbone, did not exhibit HI activity. However, HI activity emerged when the IgG backbone of clone F11 was converted to IgA1 or IgA2m2 (Fig 1A and Table 1), suggesting that anti-HA stalk IgA blocks the RBS of HA. Emergence of HI activity after conversion to IgA was confirmed by testing clone FI6 against viruses of the H1N1 subtype. The increase in HI activity against some virus strains after IgA polymerization was more pronounced for IgA2m2 than for IgA1 (Fig 1A and Table 1). To determine whether the HI activity observed for anti-HA stalk IgA was due to masking of the RBS, we conducted binding competition analyses using recombinant HA, F11 antibodies, and F045-092 IgG1 (an anti-HA RBS binding bnAb) [33]. Masking of the RBS by F11 IgG1 was quantified by measuring inhibition of F045-092 binding to HA pre-treated with F11 (Fig 1B). Binding of F045-092 IgG1 to HA after addition of F11 antibodies fell as the concentration of F11 IgG1, IgA2m2 monomer, and IgA2m2 polymer increased (Fig 1C). Notably, the RBS-masking efficiency of F11 IgA2m2 monomers and polymers (as determined by the reciprocal of the binding capacity for F045-092 IgG1) was significantly higher than that of IgG1. Furthermore, the RBS-masking efficiency of anti-HA stalk IgA antibodies increased markedly after IgA polymerization (Fig 1C). These results suggest that the IgA backbone blocks the RBS more efficiently than the IgG backbone, even in the monomeric state, and that direct blockade of the RBS by the IgA Fc region could be contributing to this phenomenon. Furthermore, increasing the molecular size of anti-HA stalk IgA antibodies by polymerization led to a marked increase in the efficiency of RBS blockade, which could be the mechanism underlying increased HI activity exhibited by anti-HA stalk IgA antibodies after IgA polymerization. This increase in RBS-blocking efficiency may be caused by the large size of the HA stalk-binding polymeric IgA antibodies, which presumably mask the RBS more efficiently than monomeric IgA antibodies. On the contrary, in some cases, the HI activity of IgA1 fell significantly after polymerization (Table 1), suggesting that the effect of IgA polymerization on HI activity is regulated in a complex manner not only by RBS blockade, but also by multiple mechanisms.

### IgA Fc glycans facilitate the HA binding and anti-viral functions of anti-HA stalk IgA antibodies, which are weakened by IgA polymerization

**Fig 2:**
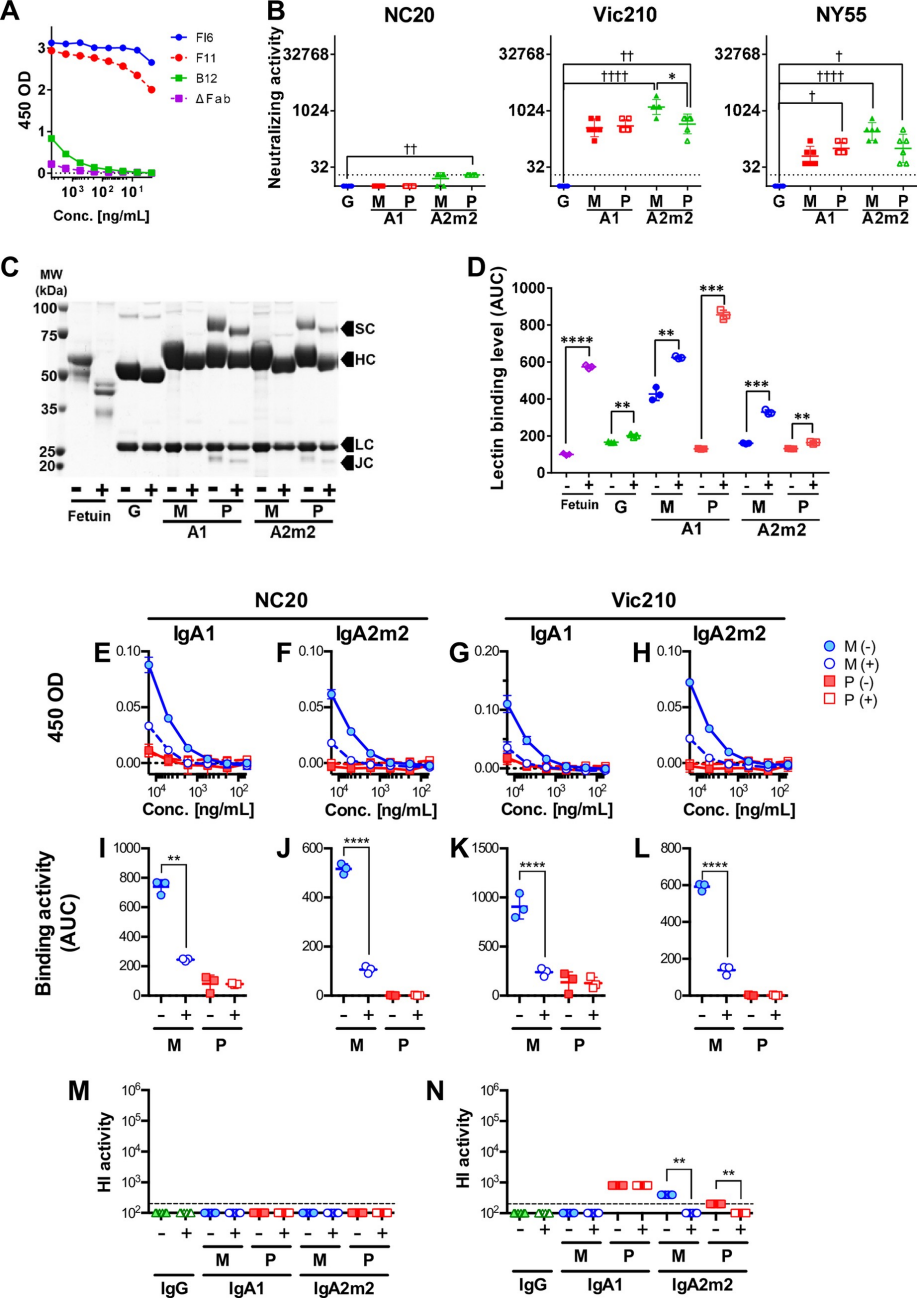
Deglycosylation of IgA antibodies and functional analyses of deglycosylated B12 IgA antibodies. (A) Reactivity of FI6, F11, B12, and ΔFab monomeric IgA1 with the recombinant A/Brisbane/59/2007 (H1N1) HA stalk. (B) Virus neutralizing (NT) activity of IgG1, monomeric (M), or polymeric (P) IgA1 (A1), and monomeric (M) or polymeric (P) IgA2m2 (A2m2) derived from antibody clone B12 against virus strains A/New Caledonia/20/1999 (H1N1, NC20), A/Victoria/210/2009 (H3N2, Vic210), and A/New York/55/2004 (H3N2, NY55). NT activity is presented in the scatter plots as the geometric mean, with the geometric standard deviation (SD) from six technical replicates. Y-axis values represent neutralizing titers, calculated as 10,000/minimum concentration (μg/ml) of antibody that neutralized the virus. Dotted lines represent the detection limit (y = 20; 500 μg/mL). *p < 0.05, comparing monomers with polymers (Mann–Whitney U test). ☨*p* < 0.05, ☨☨*p* < 0.01, ☨☨☨☨*p* < 0.0001, comparing IgA monomers/polymers with IgG (Kruskal–Wallis test, followed by Dunn’s multiple comparison test). For statistical analysis, a provisional minimum NT activity value (y = 10; 1000 μg/mL) was applied to samples in which HI activity was below the detection limit. (C) SDS-PAGE analysis of monomeric (M) and polymeric (P) B12 IgG1 (G), IgA1 (A1), and IgA2m2 (A2m2) antibodies incubated with (+) or without (-) deglycosylation enzymes. Fetuin, the band for which shifted (i.e., a reduction in molecular weight) after deglycosylation, was used as positive control. All four components of secretory IgA antibodies (heavy chain [HC], light chain [LC], secretory component [SC], and the J chain [JC]) were observed. The heavy chain, secretory component, and J chain bands of IgA shifted down slightly (reduced molecular weight) after deglycosylation (D) Deglycosylation of samples was confirmed in an enzyme-linked lectin assay (ELLA). The area under the reactivity curve (AUC) was calculated from reactivity curves for lectin and each sample was treated in the presence (+) or absence (-) of deglycosylation enzymes. Data are expressed as the mean ± SD of three technical replicates. Treatment with deglycosylation enzymes led to a significant increase in lectin binding in all samples, suggesting successful deglycosylation. **p<0.01, ***p<0.001, and ****p<0.0001 (Welch’s test). (E–H) Reactivity of glycosylated (-) and deglycosylated (+) monomeric (M) and polymeric (P) B12 IgA1 (E and G) and IgA2m2 (F and H) antibodies with recombinant HA protein from the NC20 (E and F) and Vic210 (G and H) viruses. (I–L) AUC for each glycosylated (-) and deglycosylated (+) monomeric (M) and polymeric (P) B12 IgA1 (I and K) and IgA2m2 (J and L) antibody tested against the recombinant HA protein of the NC20 (I and J) and Vic210 (K and L) viruses. The AUC was calculated from the plots in E–H. Data are expressed as the mean ± SD of three technical replicates. Deglycosylation reduced the reactivity of B12 monomeric IgA1 and IgA2m2. **p < 0.01 and ****p<0.0001 (Welch’s test). (M and N) HI titer for each glycosylated (-) and deglycosylated (+) B12 IgG1 (G), monomeric (M), or polymeric (P) IgA1 (A1) and IgA2m2 (A2m2) antibody tested against virus strains NC20 (M) and Vic210 (N). HI activity is presented in the scatter plots as the geometric mean, with the geometric SD of three technical replicates. Y-axis values represent the HI titer, calculated as the 100,000/minimum concentration (μg/ml), which represents HI activity. Dotted lines represent the detection limit (y = 200; 500 μg/mL). **p < 0.01, comparing monomers with polymers (Mann–Whitney U test). For statistical analysis, a provisional minimum HI activity value (y = 100; 1000 μg/mL) was used for samples in which the HI activity was below the detection limit.

To verify the extent to which the IgA backbone contributes to the anti-viral activity of anti-HA stalk antibodies, we examined the anti-viral functions of antibody clone B12, which is derived from gene position IGHV1-69 and lacks NT activity on an IgG backbone (S1 and S2 Tables); however, it does exhibit weak binding to the HA stalk on an IgA backbone (Fig 2A). The anti-viral and binding activities of B12 IgG1, monomeric or polymeric IgA1, and monomeric or polymeric IgA2m2 were tested against Cal7, NC20, PR8, Vic210, and NY55 viruses. The results of these assays are summarized in Table 2. Notably, binding of monomers of B12 with IgA backbones to HA was significantly higher than that of polymers. B12 IgA antibodies exhibited NT activity against certain virus strains (NC20, Vic210, and NY55), although these antibody functions did not increase after IgA1 polymerization and were weakened markedly by IgA2m2 polymerization (Fig 2B). Changes in anti-viral potency of B12 antibodies triggered by IgA polymerization tended to correspond with binding activity patterns (Table 2). This suggests that the impact of IgA polymerization has an equally negative impact on the various antibody functions of B12 IgA antibodies.

**Table 2:** Anti-viral functions of IgG and IgA antibodies derived from clones B12 and Fab-deficient (ΔFab) IgA antibodies.

		B12					ΔFab				
		H1N1			H3N2		H1N1			H3N2	
		Cal7	NC20	PR8	Vic210	NY55	Cal7	NC20	PR8	Vic210	NY55
**NT activity** [μg/mL] Geometric mean (Geometric SD)	G	>500	>500	>500	>500	>500	n.t.	n.t.	n.t.	n.t.	n.t.
	A1M	>500	>500	>500	27.8 (1.69)	158 (1.76)	n.t.	n.t.	n.t.	>500	n.t.
	A1P	>500	>500	>500	24.8 (1.43)	99.2 (1.43)	n.t.	n.t.	n.t.	>500	n.t.
	A2M	>500	630 (1.43)	>500	7.81 (1.55)	35.1 (1.69)	n.t.	n.t.	n.t.	>500	n.t.
	A2P	>500	500 (1)	>500	22.1 (1.79) *	99.2 (2.32)	n.t.	n.t.	n.t.	>500	n.t.
**HI activity** [μg/mL] Geometric mean (Geometric SD)	G	>500	>500	>500	>500	>500	n.t.	n.t.	n.t.	n.t.	n.t.
	A1M	>500	500 (1)	>500	125 (1)	158 (1.43)	n.t.	>500	n.t.	794 (1.43)	n.t.
	A1P	>500	500 (1)	>500	125 (1)	177 (1.46)	n.t.	>500	n.t.	>500	n.t.
	A2M	>500	250 (1)	>500	39.4 (1.43)	62.5 (1)	n.t.	>500	n.t.	500 (1)	n.t.
	A2P	>500	500 (1) **	>500	125 (1) **	125 (1) **	n.t.	>500	n.t.	>500	n.t.
**NI activity** [ng/mL] (EC50)	G	ND	9.57E+07	ND	1.62E+05	ND	n.t.	ND	n.t.	ND	n.t.
	A1M	ND	1.63E+05	ND	5.03E+03	1.54E+03	n.t.	ND	n.t.	ND	n.t.
	A1P	ND	5.24E+04	ND	5.05E+03	1.64E+03	n.t.	ND	n.t.	ND	n.t.
	A2M	ND	8.14E+03	ND	2.70E+03	866	n.t.	ND	n.t.	ND	n.t.
	A2P	ND	8.48E+04	ND	6.50E+03 ***	ND **	n.t.	ND	n.t.	ND	n.t.
**Binding activity** [ng/mL] (EC50)	A1M	3.80E+04	2.95E+04	1.68E+04	1.08E+05	6.28E+04	1.34E+04	2.00E+04	1.69E+04	3.65E+05	2.85E+05
	A1P	4.89E+11 ****	6.28E+10 ****	3.11E+05 ***	4.14E+05 ***	6.01E+05 **	ND ****	1.44E+10 ****	1.32E+08	8.92E+04	6.57E+06
	A2M	2.73E+04	3.08E+04	4.08E+04	2.38E+04	2.92E+04	984	1.12E+03	724	778	823
	A2P	5.28E+08 ****	3.18E+06 ****	1.18E+07 ****	1.45E+06 ****	8.44E+05 ****	2.19E+05 ****	5.98E+05 ****	6.72E+05 ****	2.51E+05 ****	1.07E+05 ****

NT activity, virus-neutralizing activity. Minimum virus-neutralizing concentrations are presented as the geometric mean (geometric SD) of six technical replicates. HI activity, hemagglutination inhibition activity. Minimum HI concentrations are presented as the geometric mean (geometric SD) of six technical replicates. NI activity, neuraminidase inhibition activity. EC_50_ values were calculated from three technical replicates. Binding activity, antibody binding activity to recombinant HA measured by ELISA. EC_50_ values were calculated from three technical replicates. G, IgG1; A1M, IgA1 monomer; A1P, IgA1 polymer; A2M, IgA2m2 monomer; A2P, IgA2m2 polymer; Cal7, A/California/7/2009 (H1N1); NC20, A/New Caledonia/20/1999 (H1N1); PR8, A/Puerto Rico/8/1934 (H1N1); Vic210, A/Victoria/210/2009 (H3N2); NY55, A/New York/55/2004 (H3N2, NY55); ND, not detected; n.t., not tested.

*p < 0.05

**p < 0.01

***p < 0.001

****p < 0.0001 (Mann–Whitney test comparing the NT and HI activity of monomers with polymers; two-way ANOVA comparing NI and binding activity curves of monomers with those of polymers).

A recent report suggests that IgA antibodies bind non-specifically to the RBS of HA via a sialylated C-terminal tail unique to IgA antibodies [34]. Therefore, we suspected that glycans on the IgA Fc region may contribute to IgA backbone-mediated binding to HA. HPLC coupled with mass spectrometry (LC-MS) analysis of N-linked glycans released from human nasal IgA and by recombinant F11 IgG1, IgA1, and IgA2m2 antibodies was conducted. In agreement with a previous report that used IgA isolated from human saliva, we detected terminally sialylated complex glycans in peaks with later retention times on the LC chromatogram for human nasal IgA. Terminally sialylated complex glycans were also detected in multiple peaks with later retention time points on the LC chromatograms for recombinant monomeric and polymeric IgA1 and IgA2m2 samples. By contrast, LC chromatograms for recombinant IgG1 showed fewer peaks than those for IgA samples, although several terminally sialylated glycans were detected in peaks with later retention times (S4 Fig). These observations suggest that both human nasal IgA antibodies and recombinant IgA antibodies contain sialic acids, and that sialylated complex glycans are more abundant in IgA than in IgG1 antibodies.

To verify whether the HA binding observed for B12 IgA was associated with glycans on the IgA Fc region, we treated B12 IgA1 and IgA2m2 monomers and polymers with a deglycosylating enzyme and compared the binding potency with that of untreated IgA (Fig 2C and 2D). We found that IgA binding to HA weakened after incubation with the deglycosylation enzyme, and that this effect was significant only for IgA monomers (Fig 2E–2L). In addition, the same deglycosylated antibody samples were tested against NC20 and Vic210 viruses in HI assays. B12 with an IgA backbone did not show any HI activity against the NC20 virus; this did not change after deglycosylation (Fig 2M). Meanwhile, B12 with an IgA backbone showed mild HI activity against the Vic210 virus, and the HI activity of B12 IgA2m2 antibodies fell after deglycosylation (Fig 2N). Taken together, these results indicate that the anti-viral activity of B12 IgA is facilitated by Fc glycan-mediated binding of IgA to HA, and that this Fc glycan-mediated effect is more pronounced for monomers than for polymers.

### IgA Fc glycan-mediated binding to the HA head contributes to anti-viral functions in the presence of Fab paratope-mediated binding to the HA stalk

**Fig 3:**
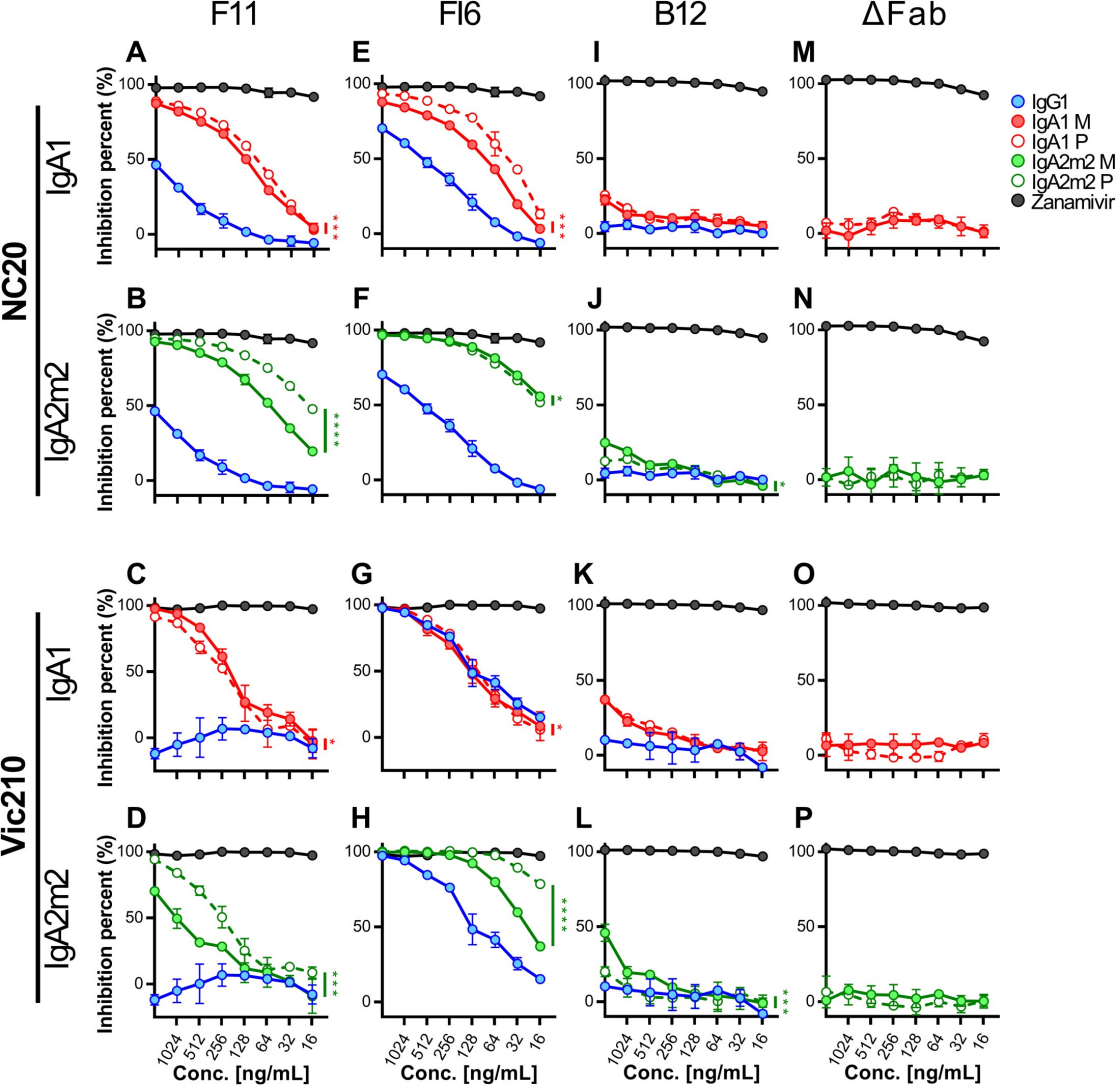
Comparison of the neuraminidase inhibition activity of IgG with that of IgA monomers and polymers. Neuraminidase inhibition (NI) activity of IgG1, monomeric (M) or polymeric (P) IgA1, and monomeric (M) or polymeric (P) IgA2m2 derived from antibody clones F11 (A–D), FI6 (E-H), and B12 (I–L), as well as Fab-deficient (ΔFab) IgA antibodies (M–P), against virus strains A/New Caledonia/20/1999 (H1N1, NC20) (A, B, E, F, I, J, M, and N) and A/Victoria/210/2009 (H3N2, Vic210) (C, D, G, H, K, L, O, and P). NI activity is presented as inhibition curves, with each point and error bar representing the mean ± SD of three technical replicates. Y-axis values represent percentage inhibition of neuraminidase activity. The OD values of wells incubated without antibodies were normalized to y = 100, and the OD values of wells incubated without virus were normalized to y = 0. X-axis values represent the concentration of antibody added to each well. Zanamivir, a neuraminidase inhibitor, was used as a positive control. *p < 0.05, **p < 0.01, ***p < 0.001, and ****p < 0.0001, comparing monomers with polymers (two-way ANOVA).

Recent reports show that anti-HA stalk antibodies exert NI activity via steric hindrance of neighboring NA molecules, and that this activity correlates well with the *in vitro* NT activity and *in vivo* anti-viral activity of anti-HA stalk antibodies [26–28]. Since polymeric IgA antibodies are larger than monomers, we assumed that IgA polymers would show higher NI activity than IgG and IgA monomers. We tested this hypothesis by conducting a NI-ELLA (Enzyme-Linked Lectin Assay) using F11, FI6, B12 IgG, and IgA antibodies. In addition, we tested the NI activity of monomeric and polymeric forms of Fab-deficient (ΔFab) IgA1 and IgA2m2 antibodies (S3C and S3D Fig) to further verify the anti-viral functions of the IgA Fc region. Both F11 and FI6 IgG showed moderate NI activity against H1N1 viruses (Fig 3A, 3B, 3E and 3F), whereas FI6 IgG showed even stronger NI activity against H3N2 viruses (Fig 3G and 3H). By contrast, F11 IgG did not show NI activity against H3N2 viruses (Fig 3C and 3D). As reported previously [26], there was a clear relationship between the NT activity and NI activity of anti-HA stalk IgG antibodies (Table 1). Interestingly, the NI activity of F11 against H3N2 viruses emerged after conversion of the antibody backbone from IgG to IgA (Fig 3C and 3D). Overall, IgA antibodies showed higher NI activity than IgG antibodies (the exception was FI6 IgA1 antibodies against H3N2 viruses) (Fig 3A–3H); in addition, the impact of IgA polymerization on NI activity differed according to the antibody clone, IgA subclass, and virus strain. Notably, B12 IgG showed minimal NI activity against the NC20 and Vic210 viruses, and B12 IgA antibodies also showed higher NI activity than B12 IgG, without IgA polymerization-induced functional enhancement (Fig 3I–3L). By contrast, ΔFab IgA antibodies did not show evident NI activity despite exhibiting HA binding activity similar to that of B12 IgA antibodies (Fig 3M–3P and Table 2). The results of these assays using ΔFab and B12 IgA antibodies suggest that the IgA Fc glycan binds to the HA RBS, leading to anti-viral activity if there is co-occurrence of Fab paratope-mediated binding to the HA stalk. Furthermore, IgA Fc glycan-mediated functions tended to fall after polymerization, indicating that, unlike Fab paratope-mediated HA binding and anti-viral activity, polymerization has a negative effect on anti-viral activity in a situation where Fab paratope-mediated HA stalk-binding is relatively weak and IgA Fc glycan-mediated HA RBS binding is predominant.

### *In vivo* passive transfer of recombinant IgG and IgA antibodies to mice

**Fig 4:**
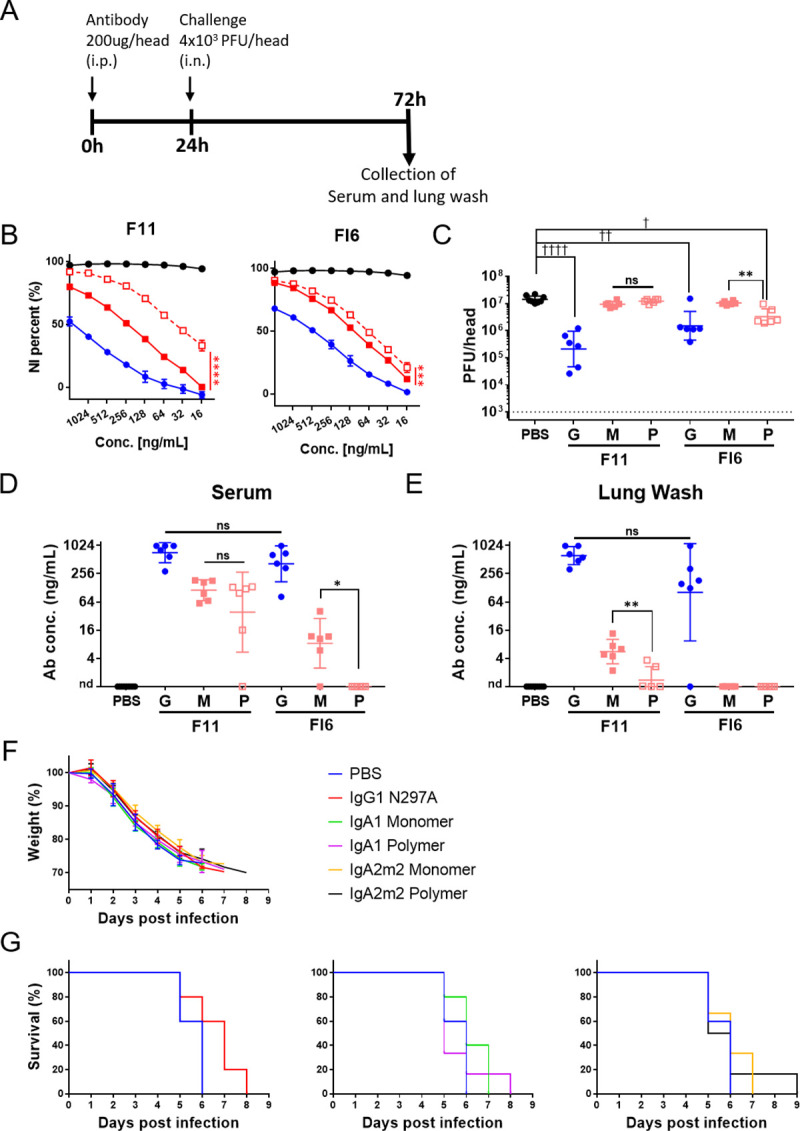
In vivo passive transfer of recombinant IgG and IgA antibodies to mice. (A) Experimental schedule. Six-week-old female BALB/c mice (six per experimental condition) were injected intraperitoneally with IgG1 (G) or IgA1 monomers (M), with polymers (P) of F11 or FI6 antibodies (200 μg/200 μl per mouse), or with PBS (200 μl per mouse) 24 hours prior to challenge with virus. Next, mice received 4×10^3^ plaque-forming units (PFU) of mNRT virus administered intranasally via the left nostril (20 μl per mouse) to infect the lower respiratory tracts. At 3 days post-infection, lung wash samples were collected. (B) NI activity of IgG1 and monomeric or polymeric IgA1 derived from antibody clones F11 and FI6 against virus strain A/California/7/2009 (H1N1). NI activity is presented as inhibition curves, with each point and error bar representing the mean ± SD of three technical replicates. Y-axis values represent percentage inhibition of neuraminidase activity. The OD values for wells incubated without antibodies were normalized to y = 100, and those for wells incubated without virus were normalized to y = 0. X-axis values denote the concentration of antibody added to each well. Black line, Zanamivir; blue line, IgG1; red solid line, IgA1 monomer; red dotted line, IgA1 polymer. ***p < 0.001 and ****p < 0.0001, comparing monomers with polymers (two-way ANOVA). (C) Virus titers in lung wash samples collected at 3 days post-infection. Virus titers are presented in scatter plots as the geometric mean, with the geometric SD. Y-axis values represent plaque-forming units (PFU)/mouse, which is equal to PFU/2mL of lung wash, as calculated from the results of the plaque assay. The dotted line denotes the detection limit (1000 PFU/mouse). **p< 0.01, comparing monomers with polymers (Mann–Whitney test). ☨*p*< 0.05, ☨☨*p*< 0.01, and ☨☨☨☨*p*< 0.0001, comparing IgA monomers/polymers with IgG (Kruskal–Wallis test, followed by Dunn’s multiple comparison test). (D, E) Antibody concentrations in serum (D) and lung wash (E) samples collected at 3 days post-infection. Antibody concentrations are expressed on scatter plots as the geometric mean, with the geometric SD. *p< 0.05 and **p< 0.01, comparing monomers with polymers (Mann–Whitney test). For statistical analysis, a provisional antibody concentration (1 ng/mL) was applied to samples in which antibody concentrations were below the detection limit. ns: not significant, nd: not detected. (F) Body weight changes in 6-week-old female BALB/c mice (five or six per experimental condition) following lethal infection with mNRT virus. Mice were injected intraperitoneally with IgG1 harboring the N297A mutation (IgG1 N297A), gA1/IgA2m2 monomers, polymers of FI6 antibodies (200 μg/200 μl per mouse), or PBS (200 μl per mouse) 2 hours prior to challenge with virus. Next, mice received 4 × 10^3^ plaque-forming units (PFU) of mNRT virus intranasally via the left nostril (20 μl per mouse) to infect the lower respiratory tract. The body weight of each mouse was normalized to that at Day 0 (the date of virus infection; y = 100). (G) Survival of mice following lethal virus infection. The survival curves for the groups receiving IgG1 N297A (left), IgA1 (middle), and IgA2m2 (right) are shown in different panels, with the survival curve of the PBS administered group used as the negative control.

Finally, to evaluate the clinical relevance of our findings in the *in vitro* experiments, we conducted an *in vivo* passive transfer experiment in which recombinant human IgG and IgA antibodies were given to mice. Mice were infected intraperitoneally with either 200 μg of IgG1, IgA1 monomer, or polymers of F11 or FI6 antibodies, 24 hours prior to challenge with mouse-adapted A/Narita/1/2009(H1N1pdm09) virus. PBS was administered to the negative control group. At 3 days post-infection, serum and lung wash samples were collected for antibody measurement and virus titration (Fig 4A). The *in vitro* anti-viral activity of F11 and FI6 IgA1 antibodies was higher than that of their IgG forms, and the NI activity of both clones was enhanced by IgA polymerization (Table 1 and Fig 4B). There was a significant decrease in lung wash virus titers in both groups receiving F11 and FI6 IgG1, suggesting that clone F11 exhibits *in vivo* anti-viral activity (Fig 4C). In the groups receiving IgA1, virus titers in lung wash from the group receiving FI6 polymeric IgA1 were significantly lower than those form the control PBS group (Fig 4C), although the concentration of FI6 IgA1 antibodies in serum and lung wash was relatively low compared with that for F11 IgA1 antibodies (Fig 4D and 4E). This suggests that higher doses of F11 IgA than FI6 IgA are required to confer anti-viral activity *in vivo*, and that the amount of F11 IgA1 administered in the current study was insufficient to neutralize or prevent efficient propagation of the virus in mice. In addition, the virus-neutralizing activity of FI6 IgA1 polymers *in vivo* was significantly higher than that of FI6 IgA1 monomers (Fig 4C). Of note, the serum FI6 IgA1 concentration in the group receiving monomeric IgA1 was significantly higher than that in the group receiving polymeric IgA1 (Fig 4D), although there was no significant difference in IgA1 concentrations in lung wash from these two groups (Fig 4E). This indicates that the observed difference in lung wash virus titers between these groups was due to differences in the *in vivo* anti-viral activity of FI6 IgA1 monomers and polymers, rather than differences in the *in vivo* stability of these two structures. In addition, FI6 IgA1, but not F11, polymers showed *in vivo* anti-viral activity, although the IgA form FI6 and F11 showed similar anti-viral activity *in vitro* and the IgG forms showed similar activity *in vivo*, suggesting that unknown factors might regulate *in vivo* anti-viral activity of the IgA forms; this is worth investigating in future studies.

Next, to measure the ability of the antibodies to protect against weight loss and mortality after lethal challenge, we conducted long-term observations of mice receiving FI6 IgG and IgA antibodies following virus infection. Of note, a previous study suggested that anti-HA stalk antibodies exert anti-viral effects via Fc receptor-mediated functions [35] and that, unlike Fc gamma receptors, mice do not express Fc alpha receptors [36]. Therefore, in this experiment, we used IgG1 harboring a N297A mutation (IgG1 N297A) [37], which inhibits the Fc-dependent anti-viral activity of anti-HA stalk IgG1 antibodies, to enable direct comparison of protection mediated by neutralizing IgG, monomeric IgA, and polymeric IgA antibodies in the absence of interference by Fc-dependent functions. We found that all mice succumbed to lethal virus challenge, regardless of the type of antibody administered, by Day 8 post-virus infection; there were no significant differences in weight loss (Fig 4F) or survival (Fig 4G) between mice receiving PBS, IgG1 N297A, or monomeric or polymeric IgA1/IgA2m2 FI6 antibodies. This suggests the importance of the Fc-dependent functions of HA stalk antibodies for protection against lethal virus infection.

## Discussion

**Fig 5:**
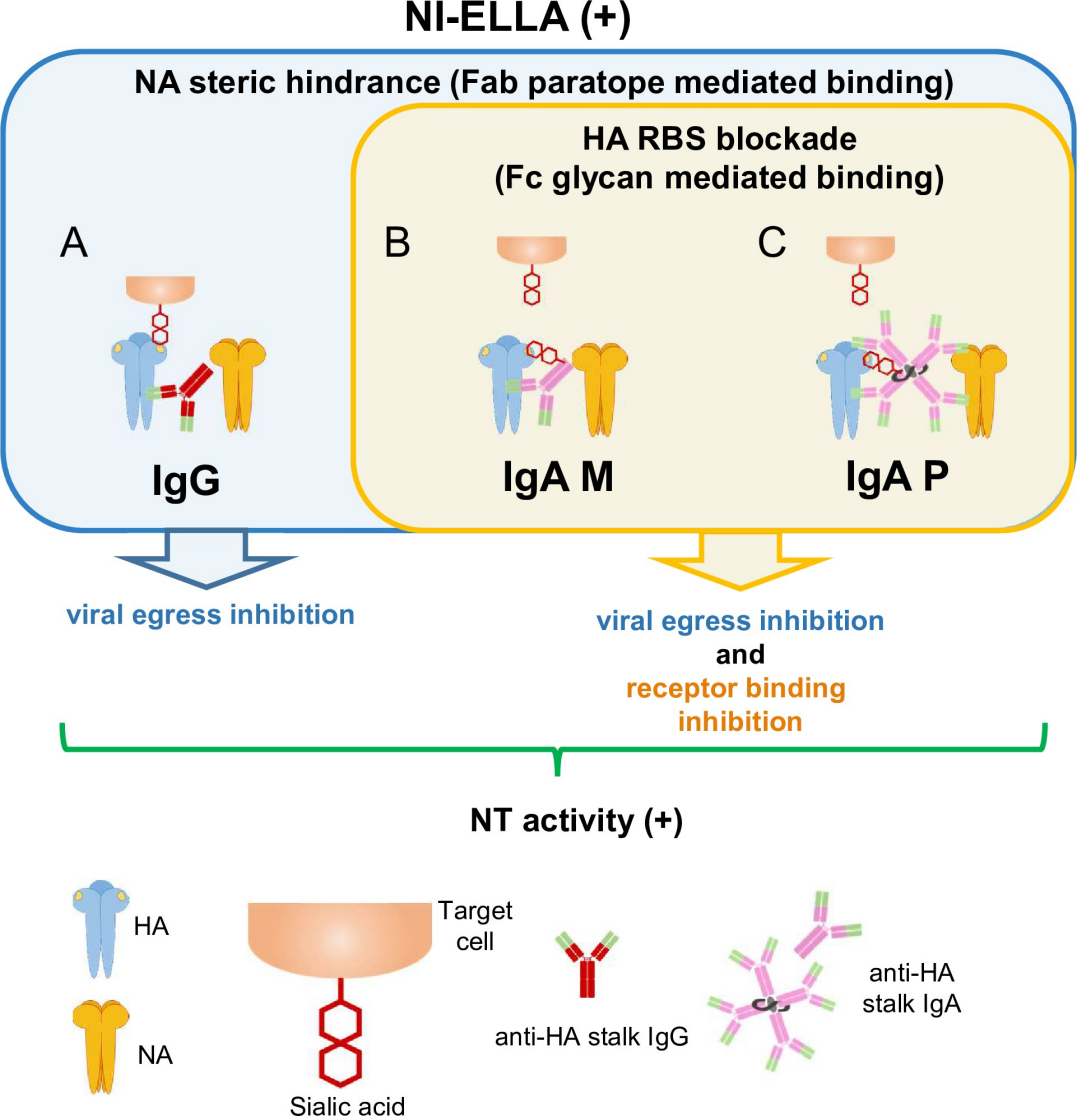
Summary of the mechanisms underlying anti-viral activity mediated by anti-HA stalk antibodies. Different modes of interaction among anti-HA stalk antibodies, NA, and HA molecules that confer anti-viral activity (including NI activity) detected in the NI-ELLA. These interactions mediate NI activity via two binding modes between antibodies and HA: Fab paratope-mediated binding to the HA stalk and Fc glycan-mediated binding to the HA head. (A) In the case of anti-HA stalk-binding IgG antibodies, Fab paratope-mediated binding to the HA stalk will cause steric hindrance of neighboring NA molecules and inhibit viral egress, leading to NT activity. (B and C) In the case of HA stalk-targeting IgA antibodies, Fc glycan-mediated binding will cause HI activity due to blockade of the receptor binding site on HA, thereby inhibiting binding of sialic acid to the receptor binding site. In these interaction modes, simultaneous steric hindrance of neighboring NA molecules and blockade of the receptor binding site may also occur, leading to inhibition of both viral egress and receptor binding.

In this study, characterization of the functional properties of IgG, monomeric IgA, and polymeric IgA antibody clones F11, FI6 (an anti-HA stalk-binding clone) [29], and B12 (a low affinity HA stalk-binding clone), as well as ΔFab IgA antibodies, revealed the impact of IgA polymerization on the functional properties of anti-HA stalk-binding antibodies (Tables 1 and 2). The data suggest that anti-HA stalk-binding IgA antibodies interact with viral glycoproteins via a mechanism different from that of anti-HA head-binding IgG antibodies. Fig 5 summarizes the possible modes of interaction between anti-HA stalk antibodies bearing anti-viral functions and viral glycoproteins, including HA and NA. Anti-HA stalk IgG antibodies bind to the HA stalk region via Fab paratopes in the absence of Fc glycan-mediated binding to the HA RBS; they then neutralize viruses by inhibiting viral egress through “NA steric hindrance”, resulting in NI activity (Fig 5A). By contrast, monomeric and polymeric anti-HA stalk IgA antibodies bind to the HA stalk region via their paratopes and to the HA RBS via Fc glycans; these antibodies sterically hinder NA and mask the HA RBS, thereby inhibiting binding of sialic acid to HA and mediating both HI activity and NI activity (Fig 5B and 5C).

SIgA plays a key role in the mechanism of action of mucosal vaccines, including intranasal inactivated influenza vaccines; indeed, one of the most prominent features of SIgA is functional enhancement due to polymerization [9–11]. A previous study using anti-HA head bnAb clone F045-092 showed that that antibody reactivity, HI activity, and virus neutralization activity improved upon IgA polymerization. Analyses revealed that these functional enhancements were most striking for viruses against which the original antibodies showed relatively low reactivity, although no evident functional enhancements were observed in the case of viruses against which the original antibodies showed relatively high reactivity [11]. A study that examined direct binding of human-derived tetrameric IgA to HA using atomic force microscopy demonstrated that HA associates with the radial regions of the IgA architecture, and that it is passed continuously from one radial region to the other [9]. Another study of human polyclonal SIgA demonstrated that the dissociation rate from HA decreased as the molecular weight of SIgA increased [10]. These results suggest that polymerization of low affinity anti-HA head antibodies increases their avidity for HA molecules, resulting in maximum functional activity. However, assays using B12, the low affinity anti-HA stalk antibody clone examined in the current study, suggests that polymerization of low affinity anti-HA stalk IgA antibodies weakens their HA binding activity and anti-viral functions. The situation is the same for ΔFab IgA antibodies, for which polymerization weakens binding to HA. Furthermore, unlike anti-HA head antibodies, no clear relationship between reactivity of anti-HA stalk F11 and FI6 IgA antibodies and other anti-viral activities (i.e., HI, NI, and NT) was observed. These observations suggest that the impact of IgA polymerization does not have an equal positive or negative impact on anti-HA stalk IgA antibody reactivity and function (i.e., NI, HI, and NT), even when the same combination of antibody clone and virus strain are tested. This indicates that IgA polymerization can either have a positive or negative effect, or no effect at all, on antibody function depending on the mode of interaction between the antibody and the viral glycoproteins. Furthermore, since NT activity is the ultimate outcome of antibody binding plus HI and NI activity, it may be that the strength of NT activity reflects the differing contributions of HA binding, HI, and NI activity. To unravel the impact of IgA polymerization on anti-viral activity, it is necessary to keep in mind that there are two types of binding module involved in the interaction between HA and IgA antibodies: specific binding between the Fab paratope and the HA stalk, and IgA Fc glycan-based binding to the HA RBS (which is the mechanism by which ΔFab IgA antibodies exert HA binding activity). These mechanisms may operate simultaneously depending on the angle or stability of the paratope and of binding to the HA stalk, thereby modulating the effect of IgA polymerization on anti-viral activity.

For example, in cases where an antibody clone can neutralize a virus strain when it is in the IgG state (i.e., lacking specific sialic acids on the antibody backbone), Fab paratope-mediated binding is dominant. In such cases, anti-viral reactivity is increased by IgA polymerization. With respect to HI activity, binding competition analyses using SPR revealed that polymeric anti-HA stalk IgA antibodies were better able to mask the RBS than monomeric anti-HA stalk IgA antibodies (Fig 1). Regarding NI activity, it can be assumed that a larger anti-HA stalk IgA antibody would more efficiently hinder neighboring NA molecules. By contrast, relatively weak Fc glycan-mediated binding will occur between a low affinity anti-HA stalk antibody clone and HA, including combinations that, at least initially, do not show NT activity in the IgG state (as seen for clone B12 IgA, and the combination of F11 IgA plus H3N2 virus). The characteristic of this glycan-mediated binding (and its derivative functions) is that the intensity weakens upon IgA polymerization, possibly due to concealment of HA-targeting glycans by the polymeric structure of SIgA. Thus, we speculate that for anti-viral functions in which intensity patterns correlate negatively with IgA polymerization, IgA Fc glycan-mediated binding dominates over paratope-dependent binding in some cases. For example, the fall in the HI and NI activities of F11 IgA1 against H3N2 viruses after polymerization (Table 1) indicates that Fc glycan-mediated binding is the main binding mode responsible for emergence of the HI, NI, and binding activity of F11 IgA1 against H3N2 viruses. NT activity was not altered markedly by IgA1 polymerization, indicating that not only Fc glycan-mediated binding but also Fab paratope-mediated binding of F11 IgA1 plays a role in neutralizing the H3N2 virus (Table 1). By contrast, the anti-viral activity of F11 IgA2m2 against H3N2 viruses clearly increased upon IgA polymerization (Table 1), suggesting that, in the case of IgA2m2, the mode of binding responsible for emergence of anti-viral functions against H3N2 viruses is Fab paratope-mediated binding.

This difference in usage of the two types of binding module between IgA1 and IgA2m2 antibodies is probably due to structural differences. IgA antibodies are abundant in mucosal excretions. Supposedly for this reason, IgA2 antibodies lack the 13-amino acid region recognized by the IgA1 proteases used by some bacterial species to evade immune responses [38]. Therefore, IgA2m2 antibodies possess shorter “arms” than IgA1 antibodies, resulting in greater accessibility to the HA stalk region, which in turn may make Fab paratope-mediated binding more favorable than is the case for IgA1. This is consistent with results reported by a study of anti-HA head antibodies in which a tetrameric IgA2m2 antibody showed markedly higher reactivity against HA proteins from all viruses than did monomeric IgA2m2 or IgA1 antibodies [11]. The observation that the positive effect of IgA polymerization on antibody reactivity is more evident for IgA2m2 antibodies supports the idea that, at least for anti-stalk IgA2m2 antibodies, the main binding module is the paratope rather than IgA Fc glycans. To summarize, in situations in which Fab paratope-mediated binding to the HA stalk exceeds IgA Fc glycan-mediated binding to HA RBS, IgA polymerization increases anti-viral functions. By contrast, when IgA Fc glycan-mediated binding to HA RBS is predominant, anti-viral activity will fall upon IgA polymerization. The coordination between these two independent binding modules (Fab paratope- and IgA glycan-mediated binding) will influence whether IgA polymerization has a negative or positive impact on the anti-viral functions of IgA molecules.

Our first *in vivo* passive transfer experiment in which mice received recombinant IgG and IgA antibodies revealed that clone F11 exhibits *in vivo* anti-viral activity in the IgG state, but not in the IgA state, possibly due to the fact that anti-HA stalk antibodies exert anti-viral effects via Fc receptor-mediated functions [35] and that, unlike Fc gamma receptors, mice do not express Fc alpha receptors [36]. The observation that FI6 IgG1 N297A did not protect mice from lethal virus challenge in the second *in vivo* passive transfer experiment further supports this idea (Fig 4G). In the absence of Fc-dependent functions, neither IgG nor IgA anti-HA stalk antibodies protected mice from lethal virus challenge, despite the finding that anti-HA IgA antibodies show higher anti-viral activity *in vivo* than IgG antibodies (Fig 4B), and despite the significant decrease in virus titer in lung wash from mice receiving FI6 IgA1 compared with the lung wash from the group receiving PBS (Fig 4C).

In conclusion, we show here that anti-HA stalk-binding IgA antibodies exert virus-neutralizing activity via two distinct modes: Fab paratope binding to the HA stalk and Fc glycan binding to the HA RBS. We also show that IgA polymerization affects the mode of antibody-mediated neutralization depending upon the affinity of the HA stalk-binding antibody. Further studies utilizing more sophisticated methods (i.e., Fc alpha receptor transgenic mice) are needed to further explore the mechanisms underlying the *in vivo* anti-viral activity of IgA, and the impact of polymerization on its function.

## Materials and methods

### Ethics statement

This non-interventional study was performed using residual peripheral blood mononuclear cells collected from nine vaccinated healthy adult volunteers participating in previous interventional clinical trials for an intranasal inactivated influenza vaccine (Clinical trial registry: UMIN000008279 [39]). The study was conducted with the approval of the medical research ethics committee of the National Institute of Infectious Diseases for the use of human subjects, Tokyo, Japan (no. 920), and all participants provided written informed consent via an ethics committee-approved form. The primary outcome of the interventional clinical trial is reported separately [39]. Participants in the interventional clinical trial received three intranasal vaccinations with a monovalent WIV vaccine (45 μg HA/500 μl). Vaccines were prepared from a reassortant vaccine candidate, IBCDC-RG2, which is derived from A/Indonesia/5/2005 (H5N1), as previously described [40]. All vaccines (lot #FPBMW1005-D) were manufactured according to GMP guidelines and were released appropriately by the Research Foundation for Microbial Disease of Osaka University (BIKEN, Kanonji, Kagawa, Japan). Six out of nine volunteers received vaccines containing carboxy-vinyl polymer (which was added as an excipient to increase viscosity). Intranasal administration was performed by spraying 0.25 ml of vaccine into each nostril (0.5 ml total) using an atomizer (Keytron Inc., Ichikawa, Chiba, Japan). For mice experiments, 7-week-old female BALB/c mice were purchased from Japan SLC (Hamamatsu, Shizuoka, Japan). Animal studies were performed in strict accordance with the Guidelines for Proper Conduct of Animal Experiments of the Scientific Council of Japan. All animal experiments were conducted in strict compliance with animal husbandry and welfare regulations in handled in biosafety level two animal facilities according to the guidelines of the Animal Care and Use Committee of the National Institute of Infectious Diseases, and were approved by this Committee (approval no. 118088). Mice were monitored daily for clinical signs of morbidity and mortality up to 14 days post infection. The human endpoint was used for mice that lost 30% or more of their initial body weight during the study. When the animals met the criteria, they were scored dead and euthanized under excess isoflurane anesthesia according to institutional guidelines.

### Viruses and cells

Madin-Darby canine kidney (MDCK) cells (American Type Culture Collection; CCL-34) were maintained at 37°C/5% CO_2_ in minimum essential medium (MEM; Life Technologies, Carlsbad, California, U.S.A.) containing 10% fetal bovine serum (Thermo Fisher Scientific, Grand Island, New York, U.S.A.) and pen-strep mix (100 units/ml penicillin and 100 μg/ml streptomycin; Life Technologies). Expi293F cells (Thermo Fisher Scientific) were maintained at 37°C/8% CO_2_ in Erlenmeyer cell culture flasks (Corning, Tewksbury, Massachusetts, U.S.A.) containing Expi293 Expression Medium (Thermo Fisher Scientific). A/California/7/2009 (H1N1)pdm09 (X-179A), A/Puerto Rico/8/1934 (H1N1), A/New Caledonia/20/1999 (H1N1), A/New York/55/2004 (H3N2), A/Victoria/210/2009 (H3N2), and mouse-adapted A/Narita/1/2009 (H1N1)pdm09 viruses were grown in 10 or 11-day-old embryonated chicken eggs. A/Narita/1/2009 (H1N1)pdm09 virus and A/Narita/1/2009 (H1N1)pdm09-derived F11 escape mutants were propagated in MDCK cells.

### Analysis of WIV intranasal vaccine-induced antibody clones

Whole blood was collected from volunteers 1 week after the second vaccination, and peripheral blood mononuclear cells were isolated using Lymphoprep™ (Abbott Diagnostics Technologies AS, Oslo, Norway). Plasmablasts from nine donors were single cell sorted (gated on markers CD2-, CD3-, CD4-, IgD-, CD38+, CD19+, and CD27+) and antibody genes from each plasmablast were cloned. The variable heavy (H) and light (L) chain regions of each clone were sequenced as described previously [41]. H and L chain sequences were subjected to analysis using IgBLAST and aligned with H and L chain variable regions of germline genes. The variable region germline genes of origin (V, D, and J for the H chain; V and J for the L chain), the length of the three CDRs, and degree of homology with the germline gene of each antibody clone were analyzed.

### Expression and purification of IgG and IgA antibodies

Plasmids encoding the α1H, α2H, human JC, and human SC were prepared as previously described [11]. To generate Fab-deficient (ΔFab) IgA1 and IgA2m2 antibodies, plasmids encoding α1H and α2H, comprising regions from CH2 to the C-terminus, were used [42]. Human SC lacking a thrombin recognition site and harboring a hexa-histidine affinity tag (His tag) at the C-terminus was synthesized and cloned into the pCXSN vector. DNA fragments encoding the variable region of the H or L chain of each clone were amplified by PCR using cDNA obtained from single-sorted plasmablasts from vaccinated healthy adult volunteers. DNA fragments encoding the variable region of the H or L chain of antibody clone FI6 were *Homo sapiens* codon-optimized and synthesized by the GeneArt Strings DNA Fragments service. Synthesized DNA fragments were cloned into α1H, α 2H, γ1HC, or κLC vectors. For expression of the IgG1 N297A mutant, the N297A mutation was introduced into the FI6 γ1HC vector by standard site directed mutagenesis and inverse PCR. To generate IgG1, Expi293F cells grown in Expi293 Expression Medium were diluted to 2.5 × 10^6^ cells/ml and transfected with 50 μg of γ1HC and 50 μg of LC per 100 ml of final culture volume using the ExpiFectamine 293 Transfection Kit (Thermo Fisher Scientific). To generate monomeric IgA, Expi293F cells were transfected with 50 μg of αH and 50 μg of LC per 100 ml of final culture volume. To generate polymeric SIgA, Expi293F cells were transfected with 80 μg of αH, 80 μg of LC, 40 μg of JC, and 40 μg of SC per 200 ml of final culture volume. At 7 days post-transfection, cell culture supernatants were centrifuged at 1200 × g and filtered to remove cell debris. The supernatants were then purified using CaptureSelect IgG-Fc (Hu) (Thermo Fisher Scientific) or CaptureSelect IgA (Thermo Fisher Scientific), according to the manufacturer’s instructions. Polymeric SIgA used for ELISA, NT, and HI assays was prepared by expressing SIgA plus the SC with a His tag, followed by purification with CaptureSelect and separation of polymeric IgA from monomeric IgA by His tag affinity chromatography using a HisTrap excel column (GE Healthcare Bio-Sciences, Uppsala, Sweden). Polymeric SIgA used for the SPR assay was prepared by expressing IgA with the SC and His tag, followed by purification with CaptureSelect and separation by gel filtration chromatography on a Superose6 Increase 10/300 GL column (GE Healthcare Bio-Sciences). Purified antibodies were concentrated using Amicon Ultracell (Merck, Darmstadt, Germany) centrifugation units with a cut-off of 30 kDa; the buffer was changed to 20 mM phosphate buffer (pH 7.4) using a Zeba Spin Desalting Column (Thermo Fisher Scientific).

### Assessment of monomeric and polymeric IgA antibody quality by SDS-PAGE and HPLC analyses

To confirm successful production and purification of monomeric and polymeric IgA antibodies, purified antibodies were analyzed by SDS-PAGE on Bolt 4–12% Bis-Tris gels (Thermo Fisher Scientific). Precision Plus Protein All Blue standards (Bio-Rad Laboratories, Inc.) were used as molecular weight markers. The SDS-PAGE gels were stained with SimplyBlue SafeStain (Thermo Fisher Scientific). Next, IgA antibodies were analyzed using the Agilent HPLC system equipped with a protein exclusion HPLC column (KW404-4F; 4.6 mm I.D. × 300 mm L; SHODEX, Japan). Samples (2 μg) were injected onto the column and separated in PBS at a flow rate of 0.2 mL/min for 34 min at 22 °C. Detection was conducted by UV at 280 nm (reference wavelength, 360 nm). Although B11, F11, and ΔFab IgA antibodies could be analyzed by HPLC, FI6 IgA samples, which tended to adhere to HPLC columns, could not. Thus, FI6 IgA samples were analyzed by sedimentation velocity analytical ultracentrifugation SV-AUC.

### SV-AUC

SV-AUC experiments were conducted using five samples each of FI6 IgG1, IgA1 monomer/polymer, and IgA2m2 monomer/polymer at 0.1 mg/mL and 1 mg/mL in 20 mM phosphate buffer with 150 mM NaCl (pH 7.4). Briefly, 390 μL of each sample was loaded into the sample sector of a cell equipped with sapphire windows and a 12 mm double-sector charcoal-filled epon centerpiece. Next, 400 μL of 20 mM phosphate buffer containing 150 mM NaCl was loaded into the reference sector of each cell. Data collection was performed at 20°C/35,000 rpm using the Optima AUC (Beckman Coulter) with a UV detection system. Data were collected every 120 s at 230 nm for each 0.1 mg/mL sample and at 290 nm for each 1 mg/mL sample, with a radial increment of 10 μm. Collected data were analyzed by the program SEDFIT (version 16.2b) [43], using continuous *c(s)* distribution fitting for the frictional ratio, meniscus, time-invariant noise, and radial-invariant noise, and a regularization level of 0.68. Sedimentation coefficient ranges of 0–30 were evaluated at a resolution of 300. The partial specific volume of samples was used as 0.73 cm^3^/g. The program SEDNTERP 1.09 [44] calculated the buffer density and viscosity as 1.0066 g/cm^3^ and 1.023 cP (Centipoise), respectively. Images of *c(s)* distribution were generated using the program GUSSI [45].

### Expression and purification of recombinant trimeric HA proteins

The mammalian cell-derived HA proteins used in this study were as follows: A/California/7/2009 (H1N1)pdm09; A/Narita/1/2009 (H1N1)pdm09 (including wild-type virus, F11 escape mutant C1 virus, and F11 escape mutant G6 virus); A/Puerto Rico/8/1934 (H1N1); A/New Caledonia/20/1999 (H1N1); A/New York/55/2004 (H3N2); A/Victoria/210/2009 (H3N2); and A/Indonesia/5/2005 (H5N1). These HA proteins comprise the extracellular domain of HA, which is C-terminally fused to the thrombin site, the trimeric Foldon of T4 fibritin, and a His tag or a Strep-tag II plus a His tag. The trimeric HA stalk of virus strain A/Brisbane/59/2007 (H1N1) was expressed as described previously [19]. HA proteins were expressed using the Expi293 Expression System (Thermo Fisher Scientific), according to the manufacturer’s instructions. At 7 days post-transfection, the medium was clarified by centrifugation at 1200 × g, filtered, and purified on a HisTrap excel column (GE Healthcare Bio-Sciences). The purified HA proteins were concentrated using Amicon Ultracell (Merck) centrifugation units with a cut-off of 30 kDa. The buffer was then changed to PBS (pH 7.4) using a Zeba Spin Desalting Column (Thermo Fisher Scientific). The HA proteins were stored at -80°C until use.

### ELISA screening to identify anti-HA stalk-binding antibody clones

Half-area flat-bottomed microtiter plates (Corning) were coated overnight at 4°C with recombinant trimeric HA proteins (50 ng/well) derived from virus strains A/California/7/2009 (H1N1)pdm09, A/Narita/1/2009 (H1N1)pdm09, A/Victoria/210/2009 (H3N2), A/Indonesia/5/2005 (H5N1), and A/Brisbane/59/2007 (H1N1)-stalk. After blocking for 1 h at 37°C with 1% BSA-PBS (pH 7.4), each monoclonal IgG1 antibody (250 ng/well) was added. Plates were incubated for 2 h at 37°C and washed three times with 0.05% PBS-Tween 20. Next, an HRP-conjugated goat anti-human IgG-Fc antibody (Bethyl Laboratories, Montgomery, Texas, U.S.A.) was diluted (1/1000) and added to each well. Plates were incubated for 1 h at 37°C, washed three times, and incubated with One-Step Ultra TMB ELISA HRP substrate solution (Thermo Fisher Scientific). The HRP reaction was stopped by addition of H_2_SO_4_, and optical density at 450 nm (OD450, reference: 655 nm) was measured in an iMark™ Microplate Reader (Bio-Rad, Hercules, California, U.S.A.).

### Preparation of F11 IgG1 fabs

F11 IgG1 antibodies were digested with immobilized papain slurry (Pierce Biotechnology, Rockford, Illinois, U.S.A.), according to the manufacturer’s instructions. Next, Fabs were purified by applying the digested antibodies to a CaptureSelect IgG-Fc (Hu) column (Thermo Fisher Scientific) and collecting the flow-through. Fabs were concentrated using Amicon Ultracell (Merck, Darmstadt, Germany) centrifugation units with a cut-off of 30 kDa. The buffer was changed to 20 mM phosphate buffer (pH 7.4) using a Zeba Spin Desalting Column (Thermo Fisher Scientific). Successful digestion and purification of Fabs were confirmed by SDS-PAGE.

### Plaque assay

A plaque assay was performed to compare the escape rate of the two mutant viruses from F11 IgG1. Briefly, 500 PFU (plaque-forming units) of wild-type, mutant C1, or mutant G6 virus were mixed with 25 μg of F11 IgG1 (final volume, 100 μl) and incubated at 37°C for 30 min. Following incubation, 100 μl of 1 × MEM was added to the virus/antibody mixture, which was then added to the wells of a 6-well plate containing a monolayer of MDCK cells. After incubation for 1 h at 37°C/5% CO_2_, each well was washed twice with PBS and overlaid with 2 ml of agar medium containing acetylated trypsin from bovine pancreas (SIGMA-ALDRICH, Saint Louis, Missouri, U.S.A.) at a final concentration of 10 μg/ml. Plates were stained with crystal violet following 2 days of incubation at 37°C/5% CO_2_.

### Generation of the F11 escape mutant virus

First, 1.0 × 10^6^ TCID_50_ (50% tissue culture infectious dose) of A/Narita/1/2009 (H1N1)pdm09 virus was mixed with 690 μg or 345 μg of F11 IgG1 (total volume, 1200 μl) and incubated at 37°C for 30 min. Next, trypsin acetylated from bovine pancreas (SIGMA-ALDRICH) was added (final concentration, 10 μg/ml), and the virus/antibody mixture (serially diluted 2-fold) was added to the wells of a 96-well plate containing a monolayer of MDCK cells. The plates were incubated at 37°C/5% CO_2_ for 6 or 7 days. Next, the culture medium was collected from wells showing cytopathic effects. For plaque purification of escape mutant virus, cell culture medium containing 100 TCID_50_ of virus was mixed with 5 μg of F11 IgG1 (final volume, 100 μl) and incubated at 37°C for 30 min. Following incubation, 100 μl of 1 × MEM was added to the virus/antibody mixture, which was then applied to the wells of a 6-well plate containing a monolayer of MDCK cells. After incubation for 1 h at 37°C/5% CO_2_, each well was washed twice with PBS and overlaid with 2 ml of agar medium containing trypsin acetylated from bovine pancreas (SIGMA-ALDRICH) (final concentration, 10 μg/ml). After incubation for 3 days under 37°C/5% CO_2_, mutant viruses were isolated from plaques formed in the wells.

### Deep sequencing of the F11 escape mutant virus

Deep sequencing was performed using MiSeq (Illumina, San Diego, California, U.S.A.)), essentially as described previously [46]. Briefly, viral RNA was extracted from virus isolates using the QIAamp Viral RNA Mini Kit (Qiagen, Venlo, Netherlands). A cDNA library was prepared from viral RNA using a NEBNext Ultra RNA Library Prep Kit for Illumina and an NEBNext Singleplex Oligos for Illumina (New England Biolabs, Ipswich, Massachusetts, U.S.A.), followed by purification using Agencourt AMPure XP (Beckman Coulter, Brea, California, U.S.A.), according to the manufacturer’s instructions. The library was sequenced with MiSeq and MiSeq Reagent Kit v2. CLC Genomics Workbench 8 (CLC bio, Aarhus, Denmark) was used to align sequence reads to the reference sequence of A/Narita/1/2009 (H1N1)pdm09.

### ELISA to assess binding of F11 IgG1 and escape mutant HA

Half-area flat-bottomed microtiter plates (Corning) were coated (250 ng/well) overnight at 4°C with recombinant HA proteins from A/Narita/1/2009 (H1N1)pdm09 wild-type virus, and F11 escape mutant C1 (harboring a T333K substitution) and G6 (harboring a G480D substitution) viruses. Plates were blocked for 1 h at 37°C with 1% BSA-PBS (pH 7.4), and F11 IgG1 samples (serially diluted 3-fold) were added to each well. Following incubation for 2 h at 37°C, wells were washed three times with 0.05% PBS-Tween 20. After addition of an HRP-conjugated goat anti-human IgG-Fc secondary antibody (Bethyl Laboratories), plates were incubated for a further 1 h at 37°C, washed three times, and incubated with One-Step Ultra TMB ELISA HRP substrate solution (Thermo Fisher Scientific). The reaction was stopped by addition of H_2_SO_4_, and optical density at 450 nm (OD450, reference: 655 nm) measured in an iMark™ Microplate Reader (Bio-Rad). Binding curves and areas under the curves (AUCs) were constructed using GraphPad Prism software (GraphPad Software, San Diego, California, U.S.A.).

### Molecular modeling of influenza virus HA trimer ectodomains

Three-dimensional models for the extracellular domains of HA trimers of A/Narita/1/2009 (H1N1) virus and two F11 escape variants harboring a T333K substitution (C1 virus) or a G480D substitution (G6 virus) in the ligand-free state were constructed using the homology modeling method and Molecular Operating Environment (MOE) (Chemical Computing Group Inc., Montreal, Quebec, Canada). The crystal structure of a HA trimer of A/California/04/2009 (H1N1) virus at a resolution of 2.6 Å (PDB code: 3LZG) was used as the modeling template. Obtained models were optimized by energy minimization using MOE. Next, an Amber10:Extended Huckel Theory (EHT) force field was implemented in MOE, which combines Amber10 and EHT bonded parameters for energy minimization.

### MD simulation of HA trimer ectodomains

HA trimer models in a ligand-free state were subjected to MD simulation, as described for HA trimers of influenza virus [47], the envelope protein of lentiviruses [48–50], and the protease of feline calicivirus [51]. The simulations were performed using the pmemd.cuda.MPI module in the Amber 16 program package [52], with the ff14SB force field [53] and the TIP3P water model for simulation of aqueous solutions [54]. A non-bonded cut-off of 10 Å was used. The lengths of bonds involving hydrogen were constrained with SHAKE, a constraint algorithm that satisfies Newtonian motion [55]. The time for all MD simulations was set to 2 fs. After heating calculations were performed for 20 picoseconds up to 310 K using the NVT ensemble, simulations were executed for 100 ns in 150 mM NaCl using the NPT ensemble (at 1 atm, 310 K).

### Calculation of RMSF

RMSF was calculated as previously described [47,49,50] to quantify the structural dynamics of molecules in the MD simulations. The RMSF of Cα atoms was calculated to obtain information about atomic fluctuations of individual amino acid residues during the MD simulations. The 15,000 snapshots, obtained from MD simulations at 70–100 ns, were used to calculate the RMSF. The average structure obtained during 70–100 ns of MD simulation was used as the reference structure for RMSF calculation. RMSF, which quantifies differences between average values and those obtained at a given time of MD simulation, was calculated using the ptraj module in Amber, a trajectory analysis tool.

### IgA binding assay

Half-area flat-bottomed microtiter plates (Corning) were coated (50 ng/well) overnight at 4°C with recombinant HA proteins derived from virus strains A/California/7/2009 (H1N1)pdm09, A/Narita/1/2009 (H1N1)pdm09, A/Puerto Rico/8/1934 (H1N1), and A/New Caledonia/20/1999 (H1N1). Plates were blocked for 1 h at 37°C with 1% BSA-PBS (pH 7.4). Next, IgA samples (serially diluted 3-fold) were added to each well. Following incubation for 2 h at 37°C, wells were washed three times with 0.05% PBS-Tween 20. After addition of an HRP-conjugated goat anti-human IgA antibody (Bethyl Laboratories) and an HRP-conjugated mouse anti-human IgA2 antibody (Abcam, Cambridge, U.K.) to detect IgA1 and IgA2m2, respectively, the plates were incubated for 1 h at 37°C, washed three times, and incubated with One-Step Ultra TMB ELISA HRP substrate solution (Thermo Fisher Scientific). The reaction was stopped by addition of H_2_SO_4_. Optical density at 450 nm (OD450, reference: 655 nm) was measured in an iMark™ Microplate Reader (Bio-Rad). Binding curves and AUCs were constructed using GraphPad Prism software (GraphPad Software).

### HI assay

HI titers were examined using an influenza virus microtitration method, as previously described, with minor modifications [56,57]. Briefly, purified antibodies (serially diluted 2-fold) were mixed with an equal volume of influenza virus diluted to 4 HA units, and then incubated for 10 min at room temperature. Next, 0.5% turkey red blood cells were added to the mixture and activity was measured after incubation for 45 min at room temperature. HI activity was defined as the reciprocal of the lowest concentration (μg/ml) of antibody that completely inhibited virus-mediated hemagglutination of red blood cells.

### Microneutralization (NT) assay

The microneutralization assay was performed using MDCK cells and 100 TCID_50_ of influenza virus, essentially as previously described [58]. Briefly, each sample (serially diluted 2-fold) was mixed with an equal volume of diluent containing influenza virus (equivalent to 100 TCID_50_) and incubated for 30 min at 37°C. This mixture was added to a monolayer of MDCK cells in the wells of a 96-well plate. Four control wells containing virus or diluent alone were included on each plate. The plates were incubated for 5 days at 37°C/5% CO_2_. All wells were observed for the presence or absence of cytopathic effects, fixed with 10% formalin phosphate buffer for more than 5 min at room temperature, and then stained with Naphthol blue black. After washing and drying, cells were solubilized with 0.1 M NaOH and absorbance (A) was read at 630 nm. The average A630 value was determined from virus-only controls (Avirus) and medium-only controls (Acell). Values >50% of the specific signal, calculated using the formula X = (Acell − Avirus)/2, were considered positive for neutralization. NT activity was defined as the reciprocal of the lowest concentration (μg/ml) of antibody at which A630 was >X.

### SPR assay of F11 Fab, and of wild-type and escape mutant virus HA proteins

The SPR assay was performed using a Biacore X100 (GE Healthcare). Recombinant trimeric HA from either A/Narita/1/2009 (H1N1)pdm09 wild-type virus, F11 escape mutant C1 (T333K), or G6 (G480D) virus with a C-terminal His tag was immobilized on the surface of Sensor Chip NTA (GE Healthcare) using the NTA reagent kit (GE Healthcare), according to the manufacturer’s instructions. Following immobilization of trimeric HA (1 μg/ml for 180 seconds), the molecular interaction with F11 Fab (2-fold serial dilutions ranging from 0.5 nM to 8 nM for wild-type virus HA and from 4 nM to 64 nM for mutant virus HA) was analyzed with a contact time of 180 s and a dissociation time of 1800 s. Sensorgrams were x- and y-axis adjusted (0 = baseline) to compare the degree of F11 Fab association and dissociation from HA.

### Assessment of RBS blockade using SPR

Recombinant trimeric HA from virus strain A/New Caledonia/20/1999 (H1N1) harboring a C-terminal His tag was immobilized on the surface of Sensor Chip NTA (GE Healthcare) using the NTA reagent kit (GE Healthcare). Following immobilization of trimeric HA (1 μg/ml for 180 s), serially diluted F11 (IgG1, IgA2m2 monomer, or IgA2m2 polymer; diluted 3-fold from 0.6 μg/ml to 48 μg/ml) was added for a contact time of 180 s and a dissociation time of 180 s. After binding of the F11 antibody to HA, F045-092 IgG1 was added at a concentration of 100 μg/ml. The contact time of F045-092 IgG1 was 180 s and the relative response (RU) at report point:binding stability (the time point immediately after the end of the F045-092 IgG1 contact time) was measured as the F045-092 IgG1 binding level. The maximum stability value was acquired by measuring the RU following F045-092 IgG1 binding to HA in the absence of F11 pre-binding. The binding ratio (%) of F045-092 IgG1 to F11 IgG1- or IgA2m2-pre-bound HA was calculated by dividing each F045-092 IgG1 binding value by the maximum stability value. The AUCs of the binding ratio curves were calculated by GraphPad Prism software (GraphPad Software).

### Neuraminidase inhibition NI-ELLA

NI-ELLA was performed as described previously [26,59,60], with minor changes. Briefly, 96-well clear flat bottom polystyrene high binding microplates (Corning) were coated for at least 24 h at 4°C with fetuin (SIGMA-ALDRICH; 25 μg/ml per 100 μl/well) in carbonate-bicarbonate buffer (pH9.6; Medicago, Uppsala, Sweden). Plates were washed three times with PBST, followed by addition of antibodies (serially diluted 2-fold) and viruses diluted in PBST/BSA, and then incubated for 18 h at 37°C. An equal amount of Zanamivir, an NA inhibitor, was used as a positive control. Following incubation, plates were washed six times with PBST. Next, lectin from Arachis hypogaea (peanut) peroxidase conjugate (SIGMA-ALDRICH; 2 μg/ml; 100 μl/well) diluted in PBST/BSA was added to the plates. Plates were incubated in the dark at room temperature for 2 h, washed three times, and incubated in the dark at room temperature for a further 10 min with One-Step Ultra TMB ELISA HRP substrate solution (Thermo Fisher Scientific). The reaction was stopped by addition of H_2_SO_4_. Optical density at 450 nm (OD450, reference: 655 nm) was measured in an iMark™ Microplate Reader (Bio-Rad). Binding curves were constructed using GraphPad Prism software (GraphPad Software).

### Deglycosylation of IgA antibodies

IgA antibodies were deglycosylated using Protein Deglycosylation Mix 2 (New England Biolabs), according to the non-denaturing reaction protocol distributed by the manufacturer. Briefly, 100 μg of IgA antibody, 5 μl of 10× Deglycosylation Mix Buffer, and 5 μl of Protein Deglycosylation Mix 2 were mixed to a total volume of 50 μl and incubated at 25°C for 30 min, followed by an overnight incubation at 37°C. Following deglycosylation, buffers were switched to 20 mM phosphate buffer (PB) (pH 7.4) using a Zeba Spin Desalting Column (Thermo Fisher Scientific). Purified antibodies were analyzed by SDS-PAGE on Bolt 4–12% Bis-Tris gels (Thermo Fisher Scientific). Precision Plus Protein All Blue standards (Bio-Rad Laboratories, Inc.) were used as molecular weight markers for SDS-PAGE. The SDS-PAGE gels were stained with SimplyBlue SafeStain (Thermo Fisher Scientific). Deglycosylation of samples was confirmed in an enzyme-linked lectin assay (ELLA). Briefly, 96-well clear flat bottom polystyrene high binding microplates (Corning) were coated for at least 24 h at 4°C with antibody samples (2 μg/ml; 100 μl/well) or fetuin (SIGMA-ALDRICH) in carbonate-bicarbonate buffer (pH9.6; Medicago). Plates were washed six times with PBST, and lectin from Arachis hypogaea (peanut) peroxidase conjugate (SIGMA-ALDRICH; 2 μg/ml; 100 μl/well), serially diluted 2-fold in PBST/BSA, was added to the plates. Plates were incubated in the dark at room temperature for 2 h, washed three times, and incubated in the dark at room temperature for a further 10 min with One-Step Ultra TMB ELISA HRP substrate solution (Thermo Fisher Scientific). The reaction was stopped by addition of H_2_SO_4_. Optical density at 450 nm (OD450, reference: 655 nm) was measured in an iMark™ Microplate Reader (Bio-Rad). Binding curves were constructed and the AUCs of the binding curves were calculated using GraphPad Prism software (GraphPad Software). Deglycosylated IgA was subjected to ELISA and HI assay, as described above.

### Glycan analysis

Human nasal IgA and recombinant IgG and IgA antibodies were subjected to glycan analysis. Human nasal IgA was purified from nasal wash concentrates using CaptureSelect IgA (Thermo Fisher Scientific), according to the manufacturer’s instructions. Purified antibodies were concentrated using Amicon Ultracell (Merck, Darmstadt, Germany) centrifugation units with a cut-off of 30 kDa. The human nasal wash concentrate samples used here were residual samples collected from a past clinical study [60]. Antibodies were deglycosylated by PNGase F (Takara Bio, Shiga, Japan) treatment. The released N-glycans were purified and labeled with 2-aminobenzamide using BlotGlyco (Sumitomo Bakelite, Tokyo, Japan), according to the manufacturer's instructions. Liquid chromatography-mass spectrometry (LC-MS) analysis was performed by Sumitomo Bakelite using a LC-MS-IT-TOF apparatus (Shimadzu, Kyoto, Japan). N-glycan structures were predicted using the GlycoMod Tool (http://web.expasy.org/glycomod/) [61].

### HPLC coupled with LC-MS

The 2-AB-labeled glycan mixture was analyzed using a Shimadzu LCMS-IT-TOF MS system equipped with an RF-20A XS fluorescence detector (Shimadzu Corp.). Separation of 2-AB glycans by normal-phase HPLC was performed on an ACQUITY UPLC® BEH Glycan 1.7 μm column (2.1 mm I.D. × 150 mm L; Waters, MA, USA) with fluorescent detection at excitation and emission wavelengths of 330 and 420 nm, respectively. Samples (1 μL) were injected onto the column, which had been equilibrated with 100% solvent B (9.9% water in acetonitrile containing 0.1% formic acid). The 2-AB glycans were separated for 50 min at 40°C on a linear gradient of 0–100% solvent A (40% acetonitrile in water containing 0.1% formic acid) at a flow rate of 0.2 mL/min. The detailed settings of the MS device were as follows: detector voltage, 2.1 kV; CDL, 200°C; heat block, 200°C; Nebulizer gas, 1.5 L/min; negative ion mode auto scan, m/z 250–2000; MS2 CID energy, 50%. According to a previous report [34] and test runs, peaks observed at later retention time points were more likely to contain terminally sialylated complex glycans; thus peaks observed at later time points were selected to estimate glycan composition.

### *In vivo* passive transfer of recombinant IgG and IgA antibodies to mice

Mouse-adapted influenza virus A/ Narita/1/2009, which was obtained after 15 passages in mice, was used as a representative A(H1N1)pdm09 virus (mNRT) for the *in vivo* experiment [62]. Six-week-old female BALB/c mice (n = 6 per experimental condition) were injected intraperitoneally with IgG1 or IgA1 monomers, or with polymers of F11 or FI6 antibodies (200 μg/200 μl per mouse), or PBS (200 μl per mouse) 24 hours prior to virus challenge. Then, the mice were received 4 × 10^3^ PFU of mNRT virus intranasally via the left nostril (20 μl per mouse) to infect the lower respiratory tract. At 3 days post-infection, serum and lung wash samples were collected. Measurement of virus titers in mice lung wash samples was performed in a plaque assay, as described by Tobita *et al*. [63]. In brief, serial (10-fold) dilutions of each sample were prepared and 200 ul aliquots of each dilution were inoculated onto MDCK cells in a 6-well plate. After incubation for 1 hour to allow for sample absorption, each well was overlaid with 2 ml of agar medium. Plates were incubated for 2 days at 37°C/5% CO_2_ and stained with crystal violet for plaque counting. The concentrations of passively transferred IgG and IgA antibodies in serum and lung wash samples were measured using a human IgG quantitation kit (Bethyl Laboratories) and a human IgA quantitation kit (Bethyl Laboratories), respectively. For long-term observations following lethal challenge with virus (undertaken to evaluate the ability of antibodies to protect against morbidity and mortality), 6-week-old female BALB/c mice (five or six per experimental condition) were injected intraperitoneally with IgG1 harboring the N297A mutation, IgA1 or IgA2m2 monomers, polymers of FI6 antibodies (200 μg/200 per mouse), or PBS (200 μl per mouse) 2 hours prior to challenge with 4 × 10^3^ PFU of mNRT virus (administered intranasally via the left nostril (20 μl per mouse) to infect the respiratory tract. Body weight was measured daily. Mice that lost >30% of their original body weight were euthanized.

### Statistical analysis

All statistical analyses were performed using the Prism statistical software package (version 7.0; GraphPad Software). An unpaired Student t-test, the Mann–Whitney test, and one-way ANOVA followed by the Kruskal–Wallis test or Welch’s test, or two-way ANOVA were used to analyze each dataset, as indicated in the figure legends. The threshold for statistical significance was set at 5% (*p* < 0.05).

## Supporting information

S1 Checklist The ARRIVE guidelines 2.0: Author checklist. (PDF)

S1 Table Genetic characteristics of antibody clones derived from intranasally-vaccinated humans. Antibodies induced by intranasal inoculation with whole inactivated influenza virus (WIV) vaccine were screened for anti-HA stalk-binding clones by examining genetic characteristics, HA binding characteristics, and virus-neutralizing characteristics. A single cell sorting protocol isolated 720 plasmablasts from nine volunteers, and 452 antibody sequences were obtained. Clones with certain genetic characteristics observed in the variable heavy (H) chains of previously reported influenza virus bnAbs were selected: eight clones were derived from the IGHV1-69 gene, 6 clones from the IGHV1-2 gene, and 11 clones possessed long complementarity determining region (CDR)3.
(DOCX)

S2 Table Virus-neutralizing activity of antibody clones (derived from intranasally-vaccinated humans) against strains A/California/7/2009 (H1N1). Recombinant IgG1 antibodies from 25 antibody clones were expressed in mammalian cells and purified. Binding to recombinant HA was tested in virus neutralization (NT) and enzyme-linked immunosorbent assays (ELISAs). None of the clones showed strong binding to HA from A/Victoria/210/2009 (H3N2) virus (Vic210). Among the 25 antibody clones, clone F11 (derived from the IGHV1-69 germline gene) showed strong binding to the HA of group one influenza viruses A/California/7/2009 (H1N1)pdm09 (Cal7) and A/Narita/1/2009 (H1N1)pdm09 (NRT). In addition, clone F11 showed strong binding to the recombinant trimeric HA stalk of virus strain A/Brisbane/59/2007 (H1N1). Other antibody clones showed moderate to low binding to HA. In contrast to the results of the HA binding assays, few antibody clones showed virus-neutralizing (NT) activity. Among all 25 clones, clone F11 showed the greatest NT activity against the Cal7 virus. Therefore, F11 was selected as a potential HA stalk-binding antibody clone induced by intranasal vaccination with an inactivated influenza vaccine.
(DOCX)

S3 Table Mutant viruses that escaped clone F11 contain mutations in the HA stalk region. To identify the area of the HA molecule that binds F11, we generated and analyzed escape mutant viruses. Passage of NRT virus in the presence of high concentrations of F11 IgG1 yielded two escape mutants C1 and G6. The C1 virus escaped neutralization by F11 IgG1 completely. By contrast, the G6 virus showed limited infectivity in the presence of F11 IgG1. Full sequencing of the escape virus genomes revealed that two synonymous and two non-synonymous mutations were introduced into the genome of the C1 virus. The two non-synonymous mutations were located in the nucleoprotein (NP) and HA genes. By contrast, only a single non-synonymous mutation was identified in the genome of the G6 virus; this was located in the HA2 region of the HA gene.
(DOCX)

S1 Fig *In vitro* characterization of escape mutant viruses of anti-HA stalk antibody candidate clone F11. Two escape mutant viruses of antibody clone F11 were obtained by serial passage of A/Narita/1/2009 (H1N1)pdm09 virus in the presence of high concentrations of F11 IgG1. (A) Escape mutant C1 exhibited higher escape efficiency than escape mutant G6. (B) Recombinant trimeric HA proteins harboring each mutation (T333K (mutant C) or G480D (mutant G)) were tested in a binding assay with F11 IgG1 to examine whether the mutations in the HA gene of escape mutant viruses affected the affinity of F11 for HA proteins. Binding of F11 IgG1 to HA harboring the T333K mutation (C1 virus) was notably lower than that to wild-type HA. By contrast, binding of F11 IgG1 to HA harboring the G480D mutation (G6 virus) was moderate when compared with that to wild-type HA. (C) The area under the reactivity curve (AUC) for F11 IgG1 in response to each recombinant HA (calculated from ELISA results for F11 IgG1 and recombinant HA). Data are expressed as the mean ± SD of three technical replicates. **p < 0.01 (Kruskal–Wallis test followed by Dunn’s multiple comparison test). (D) Surface plasmon resonance (SPR) analysis revealed that F11 had strong affinity for wild-type HA from A/Narita/1/2009(H1N1)pdm09, with a high association constant (ka [1/Ms] = 2.113 × 106) and an extremely low dissociation constant (kd (1/s) = below the measurement limit). An evident decrease in association (ka [1/Ms] = 8.541 × 103) and an increase in dissociation (kd (1/s) = 1.681 × 10–4) was observed in the presence of the T333K mutation (C1 virus). Although association of the G480D mutated virus (G6) decreased (ka [1/Ms] = 8.631 × 105), dissociation remained relatively low (kd (1/s) = 1.306 × 10–5) when compared with that for wild-type HA.
(TIF)

S2 Fig *In silico* characterization of escape mutant viruses of anti-HA stalk antibody candidate clone F11. To address the structural impact of these escape mutations in the HA stalk region, we conducted molecular dynamics (MD) simulation of the HA trimer ectodomains from the NRT, C1, and G6 viruses. (A) Flow chart showing *in silico* characterization of HA trimer ectodomain structures by MD simulation. (B) Structure analysis of HA from wild-type NRT (obtained after 100 ns of MD simulation) showed that the T333K substitution occurred near the hydrophobic groove, a target of cross-clade neutralizing antibodies, whereas the G480D substitution arose at a site away from the groove. Three-dimensional location of escape mutations T333K and G480D (light green) in the HA trimer model of A/Narita/1/2009/H1N1. Purple spheres represent amino acid residues comprising hydrophobic grooves on the HA stalk. The groove of A/Narita/1/2009/H1N1 (GenBank accession number: ACR09396) comprises I389, V392, T393, and V396 in helix A, and W365 in the fusion peptide of the HA precursor. (C) Effect of the T333K and G480D mutations on the hydrophobic grooves. Hydrophobic surface patches on the stalk regions of HA trimers after 100 ns of MD simulation were identified using the protein patch analyzer in MOE and are shown in dark green. Red sticks denote side chains on residue 333 (T333, K333, and T333 for A/Narita/1/2009/H1N1, C1, and G6, respectively). Interestingly, the T333K substitution disrupted the physical continuity of the hydrophobic surface patches in the groove, possibly due to introduction of a large positively charged side chain (left and middle panels). By contrast, G480D mutant HA maintained the continuity of the hydrophobic patches (right panel). (D) Effect of the T333K and G480D mutations on the structural dynamics of HA trimers. RMSF values, which represent atomic fluctuations in the main chains of individual amino acids, were calculated using 15,000 snapshots obtained from MD simulations of 70–100 ns. HG indicates a region between positions 389 and 396, which contains residues I389, V392, T393, and V396 in the hydrophobic groove. The numbers on the horizontal axes indicate amino acid positions in the HA precursor of A/Narita/1/2009/H1N1. Notably, both the T333K and G480D substitutions induced marked changes in fluctuation profiles around the hydrophobic groove. Two independent MD simulations reproduced these results. Taken together, the results of the experimental and *in silico* studies suggest that the hydrophobic groove of the HA stalk plays a key role in interaction with clone F11. This is consistent with previous results showing that the hydrophobic groove plays a critical role in recognition of broadly neutralizing antibodies targeting the HA stalk. The data suggest that the human antibody clone F11 is an anti-HA stalk antibody with broadly neutralizing activity.
(TIF)

S3 Fig Structural confirmation of recombinant IgA antibodies. (A–C) Chromatograms obtained by HPLC analysis of recombinant monomeric and polymeric IgA antibody clones B12 (A) and F11 (B), and of Fab-deficient (ΔFab) monomeric and polymeric IgA antibodies (C). Polymeric IgA antibody samples peaked at earlier time points than monomeric IgA antibody samples, indicating differences in molecular weight. (D) SDS-PAGE analysis of recombinant monomeric (M) and polymeric (P) F11, and of ΔFab IgA1 (A1) and IgA2m2 (A2m2) antibodies. All four components of secretory IgA antibodies (heavy chain [HC] (ΔFab HC in ΔFab IgA samples), light chain [LC], secretory component [SC], and the J chain [JC]) were observed. (E–I) Sedimentation velocity analytical ultracentrifugation (SV-AUC) was performed to determine the sedimentation coefficients (s20,w) of FI6 IgG1 (E), IgA1 monomer (F), IgA2 monomer (G), IgA1 polymer (H), and IgA2 polymer (I) in solution. The main component (s20,w = 6.7 S) of IgG1 had a sedimentation coefficient typical of an antibody monomer (E). The s20,w of the main component of the IgA1 and IgA2 monomers was 6.6 S and 7.2 S, respectively, which is similar to that of the IgG1 used as a control for this study (F and G). For the IgA1 and IgA2 polymers, multiple oligomeric species were observed (H and I). The profiles of s20,w distribution did not change by diluting the samples from 1 mg/mL (black line) to 0.1 mg/mL (red line). This indicates that oligomerization of IgA is not an association-dissociation equilibrium, at least within this concentration range.
(TIF)

S4 Fig LC chromatograms of N-linked glycans from human nasal IgA antibodies and from recombinant IgG and IgA antibodies. LC chromatograms of N-linked glycans released from human nasal IgA antibodies and recombinant (r) IgG/IgA antibodies. According to our test runs, peaks at later retention time points were more likely to contain terminally sialylated complex glycans; thus peaks observed at later time points were selected to estimate glycan composition. Peak patterns of IgA samples on LC chromatograms were similar, while those of IgG samples contained fewer peaks at later time points. Human nasal IgA samples generated the highest number of peaks at later time points. Peaks numbered 1 to 16 (red arrows) were estimated to contain terminally sialylated complex glycans. The estimated glycan compositions for each peak are shown in the left panels.
(TIF)

S1 Raw images Original images for gels. Top panel: Original image of the gel shown in Fig 2, panel C. Bottom panel: Original gel shown in S3 Fig, panel D. Images were captured by the LAS-3000 Imager (FUJIFILM) using a DIA illuminator with standard sensitivity. M: molecular weight marker. Numbers indicate the loading order within the gel. All lanes in the original gel image are included in the final figure.
(TIF)
