# Supplementary material for: An influenza HA stalk reactive polymeric IgA antibody exhibits anti-viral function regulated by binary interaction between HA and the antibody
Source: PLoS One. 2021 Jan 7;16(1):e0245244. doi: 10.1371/journal.pone.0245244 (PMC7790537; doi:10.1371/journal.pone.0245244)
Supplement: S1 Table — Antibodies induced by intranasal inoculation with whole inactivated influenza virus (WIV) vaccine were screened for anti-HA stalk-binding clones by examining genetic characteristics, HA binding characteristics, and virus-neutralizing characteristics. A single cell sorting protocol isolated 720 plasmablasts from nine volunteers, and 452 antibody sequences were obtained. Clones with certain genetic characteristics observed in the variable heavy (H) chains of previously reported influenza virus bnAbs were selected: eight clones were derived from the IGHV1-69 gene, 6 clones from the IGHV1-2 gene, and 11 clones possessed long complementarity determining region (CDR)3. (DOCX) [file pone.0245244.s002.docx]

**S1 Table. Genetic characteristics of antibody clones derived from intranasally vaccinated humans**

| Selection criterion | Clone name | Heavy chain | | | | | | | | Light chain | | | | | | |
| --- | --- | --- | --- | --- | --- | --- | --- | --- | --- | --- | --- | --- | --- | --- | --- | --- |
|  |  | Isotype | Variable region gene | | | CDR length  (amino acid) | | | Germline identity  (%) | Isotype | Variable region gene | | CDR length  (amino acid) | | | Germline identity  (%) |
|  |  |  | V | D | J | 1 | 2 | 3 |  |  | V | J | 1 | 2 | 3 |  |
| IGHV1-69 | B12 | A | 1-69*01, 1-69D*01 | 1-26*01 | 5*02 | 10 | 17 | 19 | 85.5 | L | 1-44*01 | 2*01, 3*01 | 13 | 7 | 12 | 87.8 |
|  | C1 | A | 1-69*01, 1-69D*01 | 5-12*01 | 4*02 | 10 | 17 | 15 | 84 | K | 1-39*01, 1D-39*01 | 4*02 | 11 | 7 | 9 | 83.1 |
|  | D11 | A | 1-69*09 | 6-19*01, 6-6*01, 6-25*01 | 5*02, 4*02 | 10 | 17 | 12 | 84.7 | K | 1-NL1*01 | 2*02 | 11 | 7 | 9 | 92.8 |
|  | F11 | G | 1-69*09 | 1-14*01 | 4*02 | 10 | 17 | 11 | 89.9 | K | 3-11*01 | 1*01 | 11 | 7 | 9 | 94.3 |
|  | F9 | A | 1-69*09 | 5-12*01 | 4*02 | 10 | 17 | 23 | 91.2 | K | 3-20*01 | 2*01 | 12 | 7 | 9 | 95.1 |
|  | G2 | G | 1-69*09 | 2-21*01 | 3*02, 3*01 | 10 | 17 | 19 | 88.5 | K | 3-20*01 | 5*01 | 12 | 7 | 9 | 94.6 |
|  | H10 | G | 1-69*06 | 6-25*01, 5-5*01, 5-18*01 | 5*02, 4*02 | 10 | 17 | 14 | 88 | K | 3-20*01 | 1:01 | 12 | 7 | 9 | 81.1 |
|  | H5 | A | 1-69*01, 1-69D*01 | 5-12*01 | 6*02 | 10 | 17 | 17 | 100 | L | 2-14*01 | 2*01, 3*01 | 20 | 7 | 11 | 92.7 |
| IGHV1-2 | VH1-2-1 | A | 1-2*02 | 3-10*01 | 3*02, 3*01 | 10 | 17 | 13 | 90.4 | K | 3-20*01 | 4*01 | 12 | 7 | 9 | 94 |
|  | VH1-2-2 | A | 1-2*02 | 2-21*02 | 4*02 | 10 | 17 | 14 | 91.2 | L | 1-44*01 | 3*02 | 13 | 7 | 11 | 95.9 |
|  | VH1-2-3 | A | 1-2*02 | 1-20*01 | 4*02 | 10 | 17 | 14 | 95.6 | L | 7-43*01 | 3*02 | 14 | 7 | 9 | 94.9 |
|  | VH1-2-4 | A | 1-2*02 | 2-15*01 | 4*02 | 10 | 17 | 16 | 92.5 | K | 3-20*01 | 5*01 | 12 | 7 | 9 | 92.5 |
|  | VH1-2-5 | G | 1-2*02 | 6-13*01 | 6*02 | 10 | 17 | 14 | 91.9 | K | 1D-12*01 | 4*01 | 11 | 7 | 9 | 92.3 |
|  | VH1-2-6 | G | 1-2*02, 1-2*05 | 6-19*01 | 5*01, 5*02 | 10 | 17 | 15 | 91.5 | L | 1-40*01 | 1*01 | 14 | 7 | 11 | 90.3 |
| Long CDR3 | CDR3L-1 | A | 4-34*01 | 5-12*01 | 4*02 | 10 | 16 | 21 | 97.6 | L | 2-11*01 | 2*01, 3*01, 3*02 | 14 | 7 | 9 | 96.9 |
|  | CDR3L-2 | A | 3-30*18, 3-30-5*01 | 215*01 | 4*02 | 10 | 17 | 20 | 97.6 | K | 1-39*01, 1D-39*01 | 5*01 | 11 | 7 | 8 | 98.6 |
|  | CDR3L-3 | A | 3-23D*02, 3-23*04 | 2-15*01 | 6*02 | 10 | 17 | 21 | 94.9 | K | 1-39*01, 1D-39*01 | 1*01 | 11 | 7 | 9 | 96.5 |
|  | CDR3L-4 | A | 3-7*01 | 3-10*01, 3-9*01 | 6*02 | 10 | 17 | 20 | 95.3 | L | 2-11*01 | 2*01, 3*01 | 14 | 7 | 10 | 9.2 |
|  | CDR3L-5 | A | 4-4*02 | 3-9*01 | 6*02 | 11 | 16 | 22 | 100 | L | 1-40*01 | 2*01, 3*01 | 14 | 7 | 11 | 99.7 |
|  | CDR3L-6 | A | 1-8*01 | 3-3*01 | 5*02 | 10 | 17 | 20 | 98 | L | 1-51*01 | 3*02 | 13 | 7 | 12 | 96.6 |
|  | CDR3L-7 | G | 7-4-1*02 | 2-2*02, 2-15*01, 2-2*01 | 6*02 | 10 | 17 | 21 | 91.2 | K | 3-20*01 | 1*01 | 12 | 7 | 9 | 94.8 |
|  | CDR3L-8 | A | 4-59*08 | 3-3*01 | 4*02 | 10 | 16 | 23 | 100 | L | 1-51*01 | 2*01, 3*01 | 13 | 7 | 11 | 99.7 |
|  | CDR3L-9 | A | 3-30-3*03, 3-30*04 | 4-11*01, 4-4*01 | 6*02 | 10 | 17 | 23 | 89.2 | L | 1-44*01 | 1*01 | 13 | 7 | 11 | 90.1 |
|  | CDR3L-10 | A | 3-30-3*01 | 7-27*01, 6-6*01, 1-20*01 | 6*02 | 10 | 17 | 23 | 93.6 | L | 1-44*01 | 1*01 | 13 | 7 | 11 | 93.1 |
|  | CDR3L-11 | G | 3-21*01 | 2-15*01, 3-10*02, 3-16*02 | 3*02 | 10 | 17 | 21 | 94.6 | L | 1-47*01 | 3*02 | 13 | 7 | 11 | 95.9 |

Antibodies induced by an intranasal whole inactivated influenza virus (WIV) vaccine were screened for anti-HA stalk binding clones by examining genetic characteristics, HA binding characteristics, and virus neutralizing characteristics. A single cell sorting protocol isolated 720 plasmablasts from nine volunteers, and 452 antibody sequences were obtained. Clones with certain genetic characteristics observed in the variable heavy (H) chains of previously reported influenza virus bnAbs were selected: eight clones were derived from the IGHV1-69 gene, six were derived from the IGHV1-2 gene, and 11 possessed a long complementarity determining region (CDR) 3.
