# Supplementary material for: An influenza HA stalk reactive polymeric IgA antibody exhibits anti-viral function regulated by binary interaction between HA and the antibody
Source: PLoS One. 2021 Jan 7;16(1):e0245244. doi: 10.1371/journal.pone.0245244 (PMC7790537; doi:10.1371/journal.pone.0245244)
Supplement: S2 Table — Recombinant IgG1 antibodies from 25 antibody clones were expressed in mammalian cells and purified. Binding to recombinant HA was tested in virus neutralization (NT) and enzyme-linked immunosorbent assays (ELISAs). None of the clones showed strong binding to HA from A/Victoria/210/2009 (H3N2) virus (Vic210). Among the 25 antibody clones, clone F11 (derived from the IGHV1-69 germline gene) showed strong binding to the HA of group one influenza viruses A/California/7/2009 (H1N1)pdm09 (Cal7) and A/Narita/1/2009 (H1N1)pdm09 (NRT). In addition, clone F11 showed strong binding to the recombinant trimeric HA stalk of virus strain A/Brisbane/59/2007 (H1N1). Other antibody clones showed moderate to low binding to HA. In contrast to the results of the HA binding assays, few antibody clones showed virus-neutralizing (NT) activity. Among all 25 clones, clone F11 showed the greatest NT activity against the Cal7 virus. Therefore, F11 was selected as a potential HA stalk-binding antibody clone induced by intranasal vaccination with an inactivated influenza vaccine. (DOCX) [file pone.0245244.s003.docx]

**S2 Table. Virus neutralizing activity of antibody clones (derived from intranasally vaccinated humans) against strains A/California/7/2009 (H1N1)**

|  | | Binding activity (OD_450_) | | | | Minimum neutralizing concentration (µg/mL) |
| --- | --- | --- | --- | --- | --- | --- |
|  |  | Cal7 | NRT | Vic210 | HA stalk | Cal7 |
| IGHV1-69 | B12 | 0.108 | 0.102 | 0.109 | 0.130 | >250 |
|  | C1 | 0.141 | 0.135 | 0.132 | 0.153 | >250 |
|  | D11 | 0.276 | 0.241 | 0.248 | 0.285 | 250 |
|  | F11 | 2.914 | 0.890 | 0.156 | 3.500 | 0.98 |
|  | F9 | 0.188 | 0.167 | 0.187 | 0.194 | 250 |
|  | G2 | 0.196 | 0.169 | 0.182 | 0.181 | >250 |
|  | H10 | 0.217 | 0.158 | 0.175 | 0.196 | 250 |
|  | H5 | 0.169 | 0.150 | 0.170 | 0.174 | 125 |
| IGHV1-2 | VH1-2-1 | 0.180 | 0.139 | 0.165 | 0.247 | >250 |
|  | VH1-2-2 | 0.127 | 0.085 | 0.089 | 0.171 | >250 |
|  | VH1-2-3 | 0.908 | 0.138 | 0.089 | 1.614 | >250 |
|  | VH1-2-4 | 1.991 | 0.283 | 0.094 | 3.474 | >250 |
|  | VH1-2-5 | 0.494 | 0.104 | 0.086 | 0.801 | >250 |
|  | VH1-2-6 | 0.324 | 0.187 | 0.166 | 0.469 | >250 |
| Long CDR3 | CDR3L-1 | 0.193 | 0.171 | 0.151 | 0.267 | >250 |
|  | CDR3L-2 | 0.215 | 0.104 | 0.091 | 0.325 | >250 |
|  | CDR3L-3 | 0.171 | 0.130 | 0.107 | 0.238 | >250 |
|  | CDR3L-4 | 0.146 | 0.141 | 0.108 | 0.227 | >250 |
|  | CDR3L-5 | 0.649 | 0.142 | 0.101 | 1.199 | >250 |
|  | CDR3L-6 | 0.319 | 0.149 | 0.102 | 0.504 | >250 |
|  | CDR3L-7 | 0.294 | 0.111 | 0.101 | 0.452 | >250 |
|  | CDR3L-8 | 0.341 | 0.146 | 0.122 | 0.529 | >250 |
|  | CDR3L-9 | 0.444 | 0.255 | 0.228 | 0.680 | >250 |
|  | CDR3L-10 | 0.781 | 0.308 | 0.262 | 1.261 | >250 |
|  | CDR3L-11 | 0.321 | 0.202 | 0.157 | 0.422 | >250 |
| Negative control | | 0.120 | 0.114 | 0.125 | 0.121 | >250 |

Recombinant IgG1 antibodies from these 25 antibody clones were expressed in mammalian cells, purified, and tested in virus neutralization (NT) assays and in enzyme-linked immunosorbent assays (ELISAs) to measure binding to recombinant HA. None of the clones showed high binding to HA from A/Victoria/210/2009 (H3N2) virus (Vic210). Among the 25 antibody clones, clone F11 (derived from the IGHV1-69 germline gene) exhibited strong binding to the HA of group one influenza viruses: A/California/7/2009 (H1N1)pdm09 (Cal7), and A/Narita/1/2009 (H1N1)pdm09 (NRT). In addition, clone F11 showed markedly high binding to the recombinant trimeric HA stalk of virus strain A/Brisbane/59/2007 (H1N1). Other antibody clones showed moderate to low binding to HA. In contrast to the results of the HA binding assays, few antibody clones showed virus neutralizing (NT) activity. Among all 25 clones, clone F11 showed the greatest NT activity against Cal7 virus. Therefore, F11 was selected as a potential HA stalk binding antibody clone induced by intranasal vaccination with an inactivated influenza vaccine.
