# Supplementary material for: An influenza HA stalk reactive polymeric IgA antibody exhibits anti-viral function regulated by binary interaction between HA and the antibody
Source: PLoS One. 2021 Jan 7;16(1):e0245244. doi: 10.1371/journal.pone.0245244 (PMC7790537; doi:10.1371/journal.pone.0245244)
Supplement: S3 Table — To identify the area of the HA molecule that binds F11, we generated and analyzed escape mutant viruses. Passage of NRT virus in the presence of high concentrations of F11 IgG1 yielded two escape mutants C1 and G6. The C1 virus escaped neutralization by F11 IgG1 completely. By contrast, the G6 virus showed limited infectivity in the presence of F11 IgG1. Full sequencing of the escape virus genomes revealed that two synonymous and two non-synonymous mutations were introduced into the genome of the C1 virus. The two non-synonymous mutations were located in the nucleoprotein (NP) and HA genes. By contrast, only a single non-synonymous mutation was identified in the genome of the G6 virus; this was located in the HA2 region of the HA gene. (DOCX) [file pone.0245244.s004.docx]

**S3 Table. Mutant viruses that escape clone F11 contain mutations in the HA stalk region**

| Mutant virus | Mutation site (gene) | Nucleotide change | Amino acid change |
| --- | --- | --- | --- |
| C1 | NP | A169G | I57V |
|  | HA | C867T | Synonymous |
|  | HA | C998A | T333K |
|  | MP | G543A | Synonymous |
| G6 | HA | G1439A | G480D |

To find out which area of the HA molecule binds F11, we generated and analyzed escape mutant viruses. Passage of NRT virus in the presence of high concentrations of F11 IgG1 yielded two escape viruses: C1 and G6. The C1 virus escaped neutralization by F11 IgG1 completely. By contrast, the G6 virus showed limited infectivity in the presence of F11 IgG1. Full sequencing of these escape virus genomes revealed that two synonymous and two non-synonymous mutations were introduced into the genome of the C1 virus. The two non-synonymous mutations were located in the nucleoprotein (NP) gene and the HA gene. By contrast, only a single non-synonymous mutation was identified in the genome of the G6 virus; this was located in the HA2 region of the HA gene.
