# Supplementary figures and images for: An influenza HA stalk reactive polymeric IgA antibody exhibits anti-viral function regulated by binary interaction between HA and the antibody
Source: PLoS One. 2021 Jan 7;16(1):e0245244. doi: 10.1371/journal.pone.0245244 (PMC7790537; doi:10.1371/journal.pone.0245244)

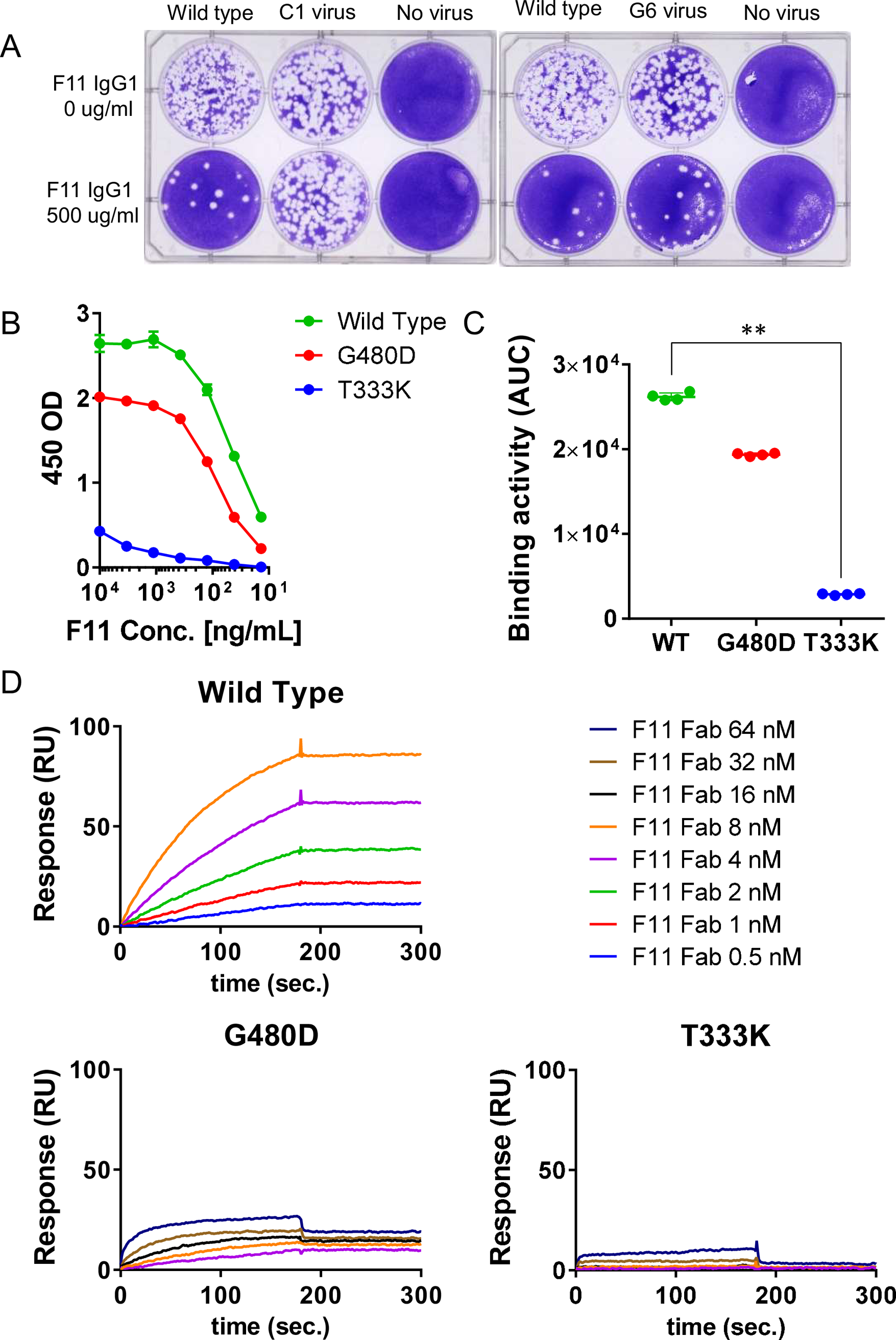

Supplement: S1 Fig — Two escape mutant viruses of antibody clone F11 were obtained by serial passage of A/Narita/1/2009 (H1N1)pdm09 virus in the presence of high concentrations of F11 IgG1. (A) Escape mutant C1 exhibited higher escape efficiency than escape mutant G6. (B) Recombinant trimeric HA proteins harboring each mutation (T333K (mutant C) or G480D (mutant G)) were tested in a binding assay with F11 IgG1 to examine whether the mutations in the HA gene of escape mutant viruses affected the affinity of F11 for HA proteins. Binding of F11 IgG1 to HA harboring the T333K mutation (C1 virus) was notably lower than that to wild-type HA. By contrast, binding of F11 IgG1 to HA harboring the G480D mutation (G6 virus) was moderate when compared with that to wild-type HA. (C) The area under the reactivity curve (AUC) for F11 IgG1 in response to each recombinant HA (calculated from ELISA results for F11 IgG1 and recombinant HA). Data are expressed as the mean ± SD of three technical replicates. **p < 0.01 (Kruskal–Wallis test followed by Dunn’s multiple comparison test). (D) Surface plasmon resonance (SPR) analysis revealed that F11 had strong affinity for wild-type HA from A/Narita/1/2009(H1N1)pdm09, with a high association constant (ka [1/Ms] = 2.113 × 10^6^) and an extremely low dissociation constant (kd (1/s) = below the measurement limit). An evident decrease in association (ka [1/Ms] = 8.541 × 10^3^) and an increase in dissociation (kd (1/s) = 1.681 × 10^–4^) was observed in the presence of the T333K mutation (C1 virus). Although association of the G480D mutated virus (G6) decreased (ka [1/Ms] = 8.631 × 10^5^), dissociation remained relatively low (kd (1/s) = 1.306 × 10^–5^) when compared with that for wild-type HA. (TIF) [file pone.0245244.s005.tif]

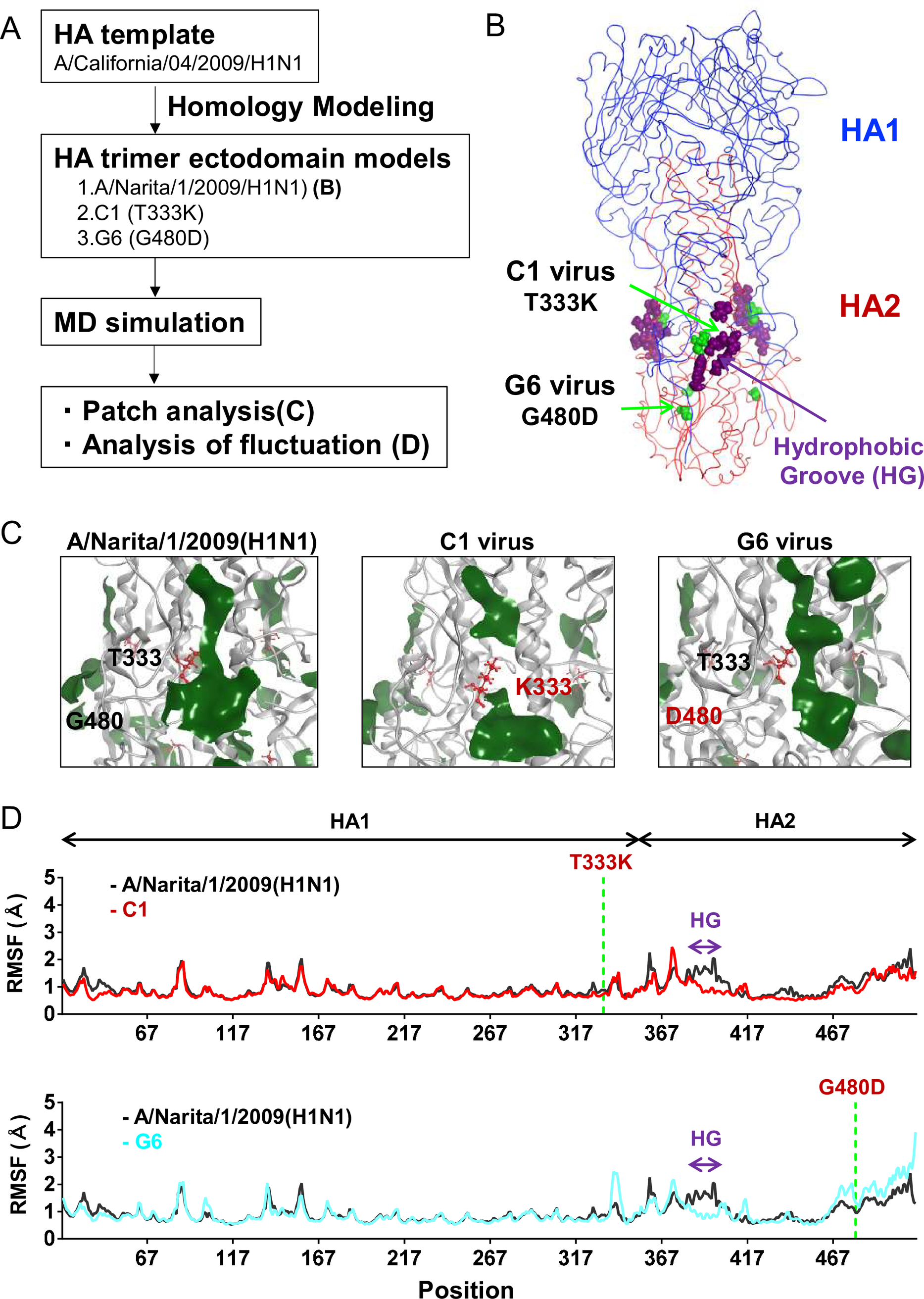

Supplement: S2 Fig — To address the structural impact of these escape mutations in the HA stalk region, we conducted molecular dynamics (MD) simulation of the HA trimer ectodomains from the NRT, C1, and G6 viruses. (A) Flow chart showing in silico characterization of HA trimer ectodomain structures by MD simulation. (B) Structure analysis of HA from wild-type NRT (obtained after 100 ns of MD simulation) showed that the T333K substitution occurred near the hydrophobic groove, a target of cross-clade neutralizing antibodies, whereas the G480D substitution arose at a site away from the groove. Three-dimensional location of escape mutations T333K and G480D (light green) in the HA trimer model of A/Narita/1/2009/H1N1. Purple spheres represent amino acid residues comprising hydrophobic grooves on the HA stalk. The groove of A/Narita/1/2009/H1N1 (GenBank accession number: ACR09396) comprises I389, V392, T393, and V396 in helix A, and W365 in the fusion peptide of the HA precursor. (C) Effect of the T333K and G480D mutations on the hydrophobic grooves. Hydrophobic surface patches on the stalk regions of HA trimers after 100 ns of MD simulation were identified using the protein patch analyzer in MOE and are shown in dark green. Red sticks denote side chains on residue 333 (T333, K333, and T333 for A/Narita/1/2009/H1N1, C1, and G6, respectively). Interestingly, the T333K substitution disrupted the physical continuity of the hydrophobic surface patches in the groove, possibly due to introduction of a large positively charged side chain (left and middle panels). By contrast, G480D mutant HA maintained the continuity of the hydrophobic patches (right panel). (D) Effect of the T333K and G480D mutations on the structural dynamics of HA trimers. RMSF values, which represent atomic fluctuations in the main chains of individual amino acids, were calculated using 15,000 snapshots obtained from MD simulations of 70–100 ns. HG indicates a region between positions 389 and 396, which contains residues I389, V [file pone.0245244.s006.tif]

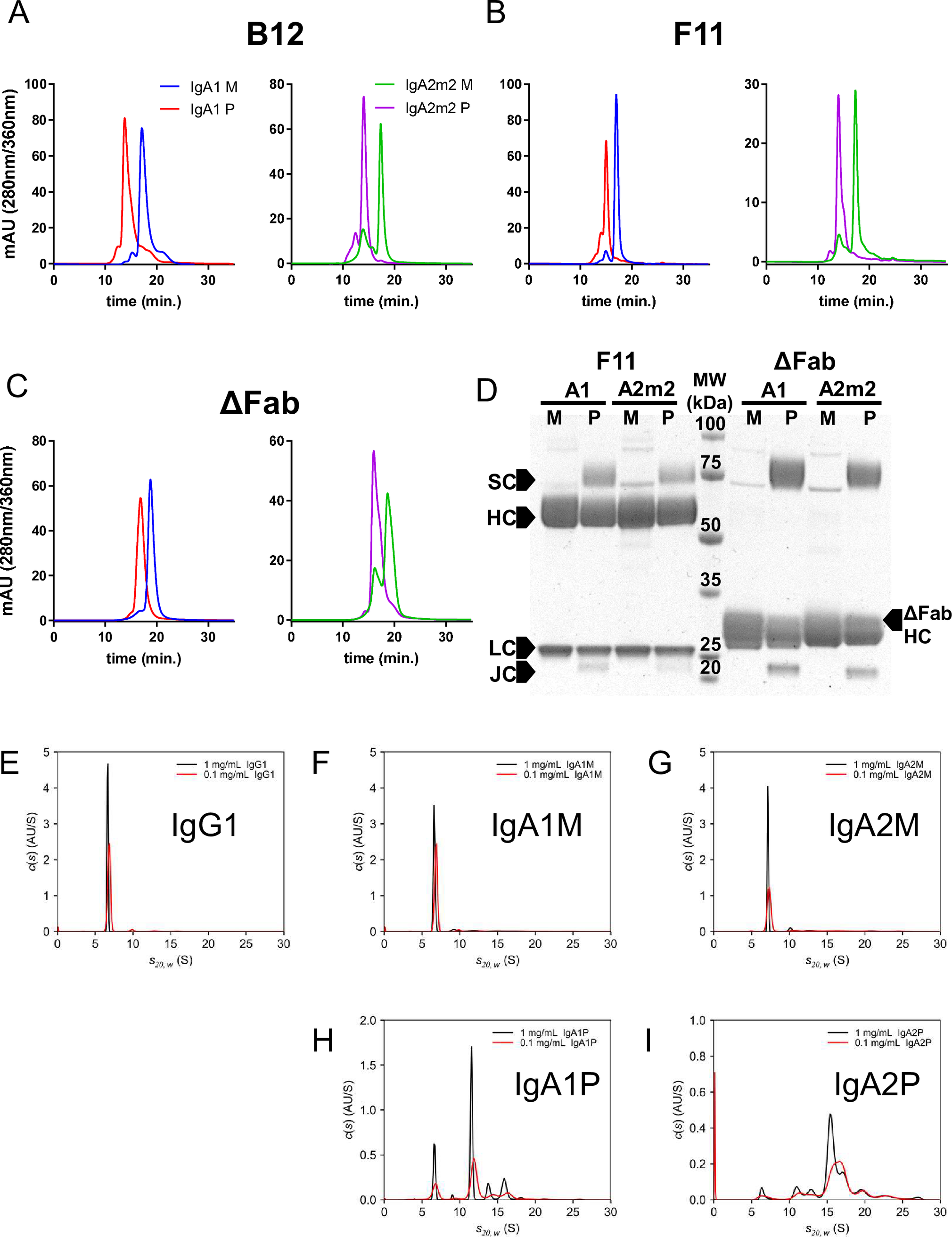

Supplement: S3 Fig — (A–C) Chromatograms obtained by HPLC analysis of recombinant monomeric and polymeric IgA antibody clones B12 (A) and F11 (B), and of Fab-deficient (ΔFab) monomeric and polymeric IgA antibodies (C). Polymeric IgA antibody samples peaked at earlier time points than monomeric IgA antibody samples, indicating differences in molecular weight. (D) SDS-PAGE analysis of recombinant monomeric (M) and polymeric (P) F11, and of ΔFab IgA1 (A1) and IgA2m2 (A2m2) antibodies. All four components of secretory IgA antibodies (heavy chain [HC] (ΔFab HC in ΔFab IgA samples), light chain [LC], secretory component [SC], and the J chain [JC]) were observed. (E–I) Sedimentation velocity analytical ultracentrifugation (SV-AUC) was performed to determine the sedimentation coefficients (s20,w) of FI6 IgG1 (E), IgA1 monomer (F), IgA2 monomer (G), IgA1 polymer (H), and IgA2 polymer (I) in solution. The main component (s20,w = 6.7 S) of IgG1 had a sedimentation coefficient typical of an antibody monomer (E). The s20,w of the main component of the IgA1 and IgA2 monomers was 6.6 S and 7.2 S, respectively, which is similar to that of the IgG1 used as a control for this study (F and G). For the IgA1 and IgA2 polymers, multiple oligomeric species were observed (H and I). The profiles of s20,w distribution did not change by diluting the samples from 1 mg/mL (black line) to 0.1 mg/mL (red line). This indicates that oligomerization of IgA is not an association-dissociation equilibrium, at least within this concentration range. (TIF) [file pone.0245244.s007.tif]

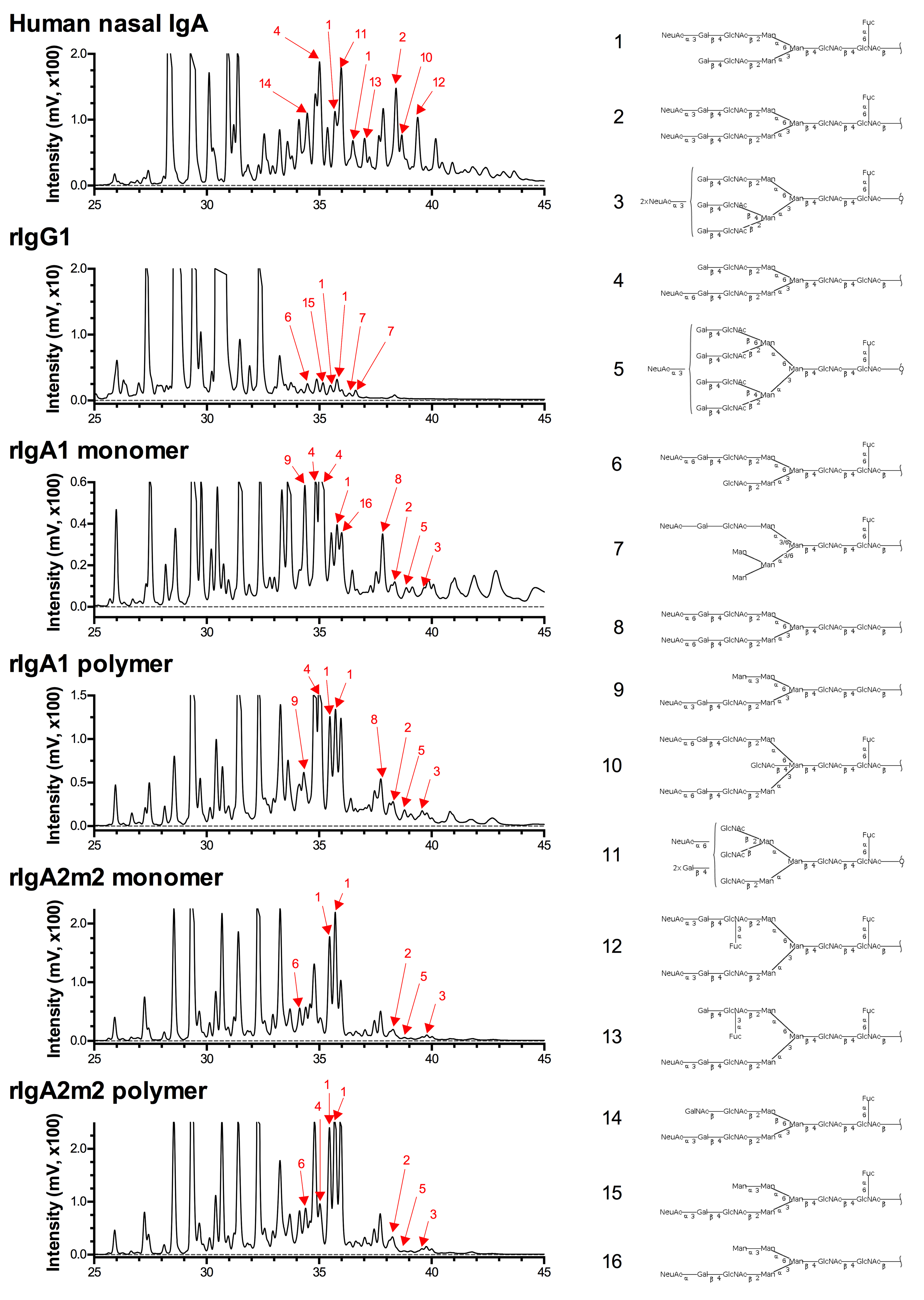

Supplement: S4 Fig — LC chromatograms of N-linked glycans released from human nasal IgA antibodies and recombinant (r) IgG/IgA antibodies. According to our test runs, peaks at later retention time points were more likely to contain terminally sialylated complex glycans; thus peaks observed at later time points were selected to estimate glycan composition. Peak patterns of IgA samples on LC chromatograms were similar, while those of IgG samples contained fewer peaks at later time points. Human nasal IgA samples generated the highest number of peaks at later time points. Peaks numbered 1 to 16 (red arrows) were estimated to contain terminally sialylated complex glycans. The estimated glycan compositions for each peak are shown in the left panels. (TIF) [file pone.0245244.s008.tif]

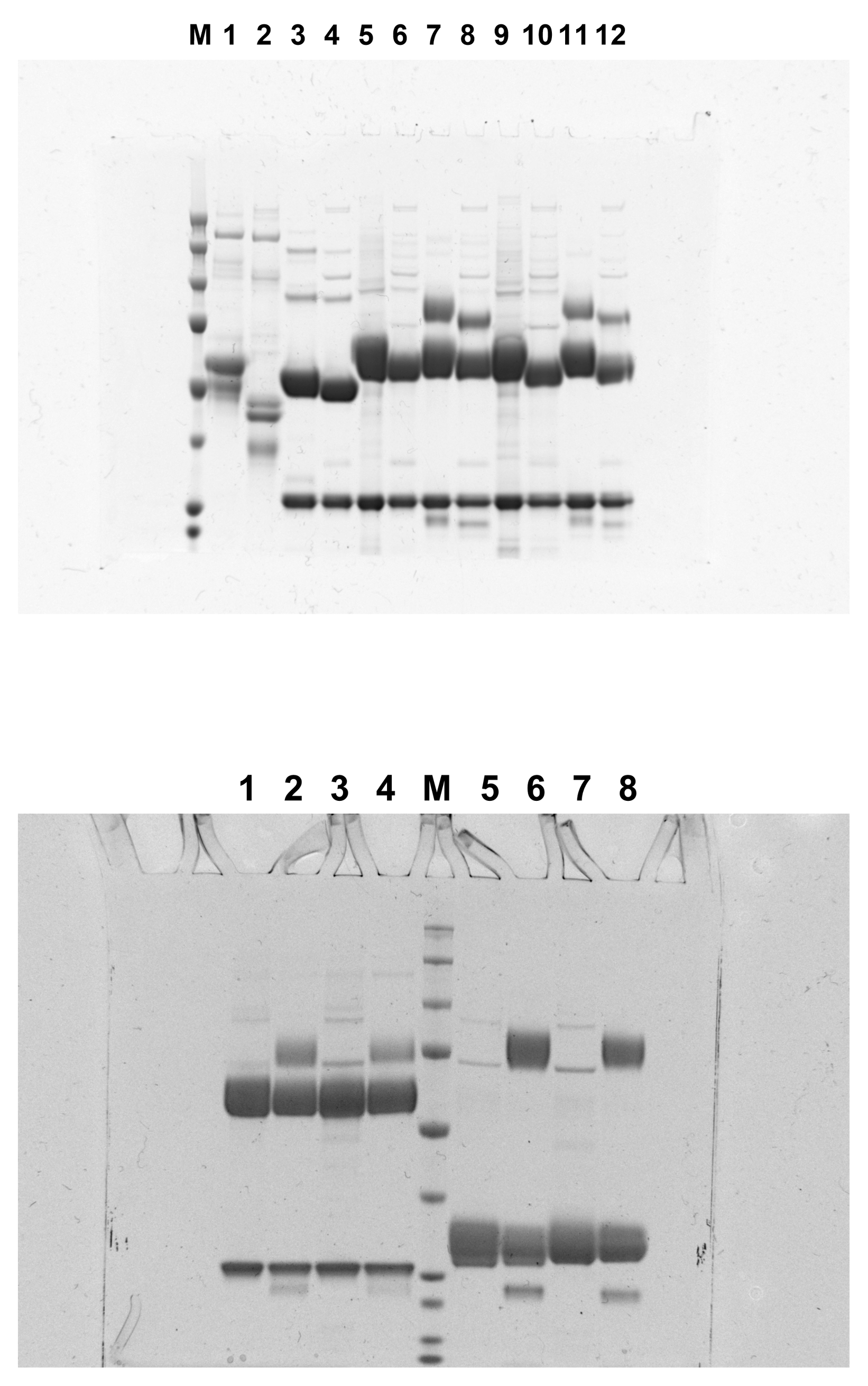

Supplement: S1 Raw images — Top panel: Original image of the gel shown in Fig 2, panel C. Bottom panel: Original gel shown in S3 Fig, panel D. Images were captured by the LAS-3000 Imager (FUJIFILM) using a DIA illuminator with standard sensitivity. M: molecular weight marker. Numbers indicate the loading order within the gel. All lanes in the original gel image are included in the final figure. (TIF) [file pone.0245244.s009.tif]
